# Molecular and electrophysiological evaluation of human cardiomyocyte subtypes to facilitate generation of composite cardiac models

**DOI:** 10.1177/20417314221127908

**Published:** 2022-10-18

**Authors:** Jiuru Li, Alexandra Wiesinger, Lianne Fokkert, Bastiaan J. Boukens, Arie O. Verkerk, Vincent M. Christoffels, Gerard J.J. Boink, Harsha D. Devalla

**Affiliations:** 1 Department of Medical Biology, Amsterdam University Medical Centers, University of Amsterdam, Amsterdam, The Netherlands; 2 Department of Experimental Cardiology, Amsterdam University Medical Centers, University of Amsterdam, Amsterdam, The Netherlands; 3 Department of Cardiology, Amsterdam University Medical Centers, University of Amsterdam, Amsterdam, The Netherlands

**Keywords:** iPSC, stem cell, cardiac differentiation, engineered heart tissues, electrophysiology

## Abstract

Paucity of physiologically relevant cardiac models has limited the widespread application of human induced pluripotent stem cell (hiPSC)-derived cardiomyocytes in drug development. Here, we performed comprehensive characterization of hiPSC-derived cardiomyocyte subtypes from 2D and 3D cultures and established a novel 3D model to study impulse initiation and propagation. Directed differentiation approaches were used to generate sinoatrial nodal (SANCM), atrial (ACM) and ventricular cardiomyocytes (VCM). Single cell RNA sequencing established that the protocols yield distinct cell populations in line with expected identities, which was also confirmed by electrophysiological characterization. In 3D EHT cultures of all subtypes, we observed prominent expression of stretch-responsive genes such as *NPPA*. Response to rate modulating drugs noradrenaline, carbachol and ivabradine were comparable in single cells and EHTs. Differences in the speed of impulse propagation between the subtypes were more pronounced in EHTs compared with 2D monolayers owing to a progressive increase in conduction velocities in atrial and ventricular cardiomyocytes, in line with a more mature phenotype. In a novel binary EHT model of pacemaker-atrial interface, the SANCM end of the tissue consistently paced the EHTs under baseline conditions, which was inhibited by ivabradine. Taken together, our data provide comprehensive insights into molecular and electrophysiological properties of hiPSC-derived cardiomyocyte subtypes, facilitating the creation of next generation composite cardiac models for drug discovery, disease modeling and cell-based regenerative therapies.

## Introduction

The difficulty in obtaining native human cardiac tissue and inter-species differences in electrophysiological properties^1^ propelled the need for alternative models that resemble cell types of the human heart for applications in disease modeling and drug screening. In this regard, human induced pluripotent stem cell (hiPSC)-derived cardiomyocytes and associated models have emerged as attractive tools. Whilst these cell types have undoubtedly proven their value in advancing our understanding of cardiac development and unraveling disease mechanisms,^2,3^ the widespread application of these models in cardiotoxicity screenings and drug discovery is still lagging behind.^4,5^ This is in part due to limitations in existing models, which do not recapitulate the intricate interrelationship of the different regions of the heart.

The coordinated and rhythmic action of distinct components of the heart such as nodal, atrial, and ventricular is central to normal cardiac physiology. The majority of the current in vitro cardiomyocyte models including 2D cultures and 3D constructs such as engineered heart tissues (EHTs)^6,7^ or microtissues^8^ focus on one individual cardiomyocyte subtype niche, typically that of ventricular cardiomyocytes (VCM). With the introduction of directed differentiation protocols, it is now possible to generate other cardiomyocyte subtypes such as atrial (ACM) and sinoatrial nodal cardiomyocytes (SANCM).^9–12^ To facilitate the creation of physiologically relevant composite models such as the pacemaker-atrial interface, an important prerequisite is a comparative assessment of molecular and functional properties of the various cardiomyocyte subtypes.

Here, we generated SANCM, ACM, and VCM from hiPSCs and assessed their gene expression characteristics to define the composition of these cultures. Next, we performed electrophysiological assessment of the three groups. Furthermore, we evaluated the response of each cardiomyocyte subtype obtained from (a) 2-dimensional (2D) monolayer cultures, and (b) 3-dimensional (3D) EHTs to noradrenaline, carbachol, and ivabradine. Lastly, we generated dual component tissues comprised of SANCM and ACM to evaluate impulse initiation and propagation in a rudimentary functional in vitro model of the pacemaker-atrial interface.

## Results

### Generating cardiomyocyte subtypes from hiPSCs

**Figure 1.**
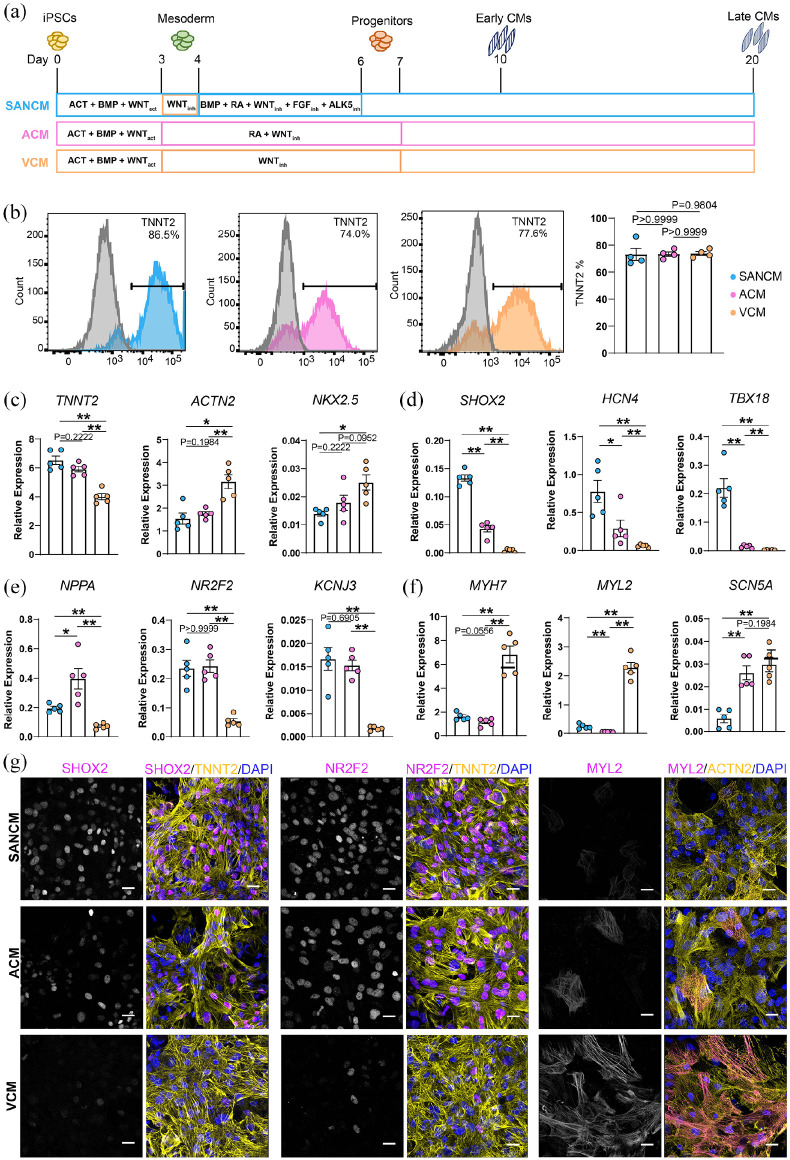
Differentiation and characterization of cardiomyocyte subtypes. (a) Schematic representation of protocols utilized for the differentiation of cardiomyocyte subtypes from hiPSCs. (b) Representative histograms and summarized data showing % of TNNT2^+^ cells in the three groups at day 20 of differentiation. A corresponding IgG isotype antibody was used as negative control for flow cytometry (gray). *n* = 4 independent differentiations. Error bars, s.e.m. Mann-Whitney *U* test. (c–f) qRT-PCR analysis demonstrating expression of cardiomyocyte genes (c), SAN-associated genes (d), atrial-associated genes (e), and ventricular-associated genes (f). *N* = 5 independent differentiations. Expression corrected to GEOMEAN of reference genes *RPLP0* and *GUSB*. Error bars, s.e.m. Kruskal Wallis Test followed by Mann Whitney *U*-test for post hoc comparison. *p < 0.05, **p < 0.01. (g) Immunofluorescence stainings demonstrating the expression of SHOX2 and TNNT2, NR2F2 and TNNT2 and, MYL2 and ACTN2, in SANCM, ACM, and VCM. Scale bars, 20 μm.

To generate cardiomyocytes, hiPSCs were coaxed toward cardiac mesoderm using cytokines and small molecules that activate Activin/Nodal, bone morphogenetic protein (BMP) and wingless-related integration site (WNT) signaling.^9,13^ At the cardiac mesoderm stage, inhibition of WNT signaling alone or in combination with activation of retinoic acid (RA) signaling was used to generate VCM and ACM, respectively. To generate SANCM, BMP, and RA signaling were activated in conjunction with inhibition of WNT, TGFβ and FGF signaling, as reported previously^12^ (Figure 1(a)). Contractile cells were typically observed in all groups around day 10 and whole syncytia were formed by day 14. Flow cytometry analysis of cardiac sarcomeric protein, TNNT2 confirmed the presence of 70%–80% cardiomyocytes in all three groups (Figure 1(b)).

To verify cardiomyocyte subtype identity, we assessed the expression of typical cardiac genes as well as genes associated with specific cardiomyocyte subtypes (Figure 1(c)–(f)) by quantitative real-time PCR (qRT-PCR) at day 20 of differentiation. Cardiac genes *TNNT2* and *ACTN2* displayed contrasting expression patterns. Whilst *TNNT2* expression was higher in SANCM and ACM, the expression of *ACTN2* was higher in VCM. We also assessed the expression of *NKX2-5*, which is expressed in working myocardial cells but is absent in a subset of SAN cardiomyocytes in vivo.^14^ Consistent with this finding, we observed lower expression of *NKX2-5* in SANCM. Nonetheless, the detection of *NKX2-5* expression in the SANCM group suggests the presence of a fraction of cells that do express this gene. Similarly, the expression of SAN-associated transcription factor *SHOX2* and ion channel gene *HCN4*, which encodes the funny current (I_*f*_), were enriched in SANCM but some expression was also detected in ACM. However, the expression of transcription factor *TBX18*, an important regulator of pacemaker cell development^14^ was exclusive to SANCM. On the other hand, atrial associated gene, *NPPA* was predominantly present in ACM and expression was also observed in SANCM. The orphan nuclear transcription factor gene, *NR2F2*, which is selectively expressed in the developing inflow tract^15^ and subsequently in both the sinoatrial node (SAN) and the atria^16–19^ was expressed similarly in SANCM and ACM. Comparably, the inward rectifying K^+^ channel gene, *KCNJ3*, which is involved in the generation of acetylcholine-activated K^+^ current (I_*KACh*_) in both the SAN and the atria^20^ was elevated in SANCM and ACM, compared with VCM. Lastly, the expression of ventricular associated genes, *MYL2* and *MYH7* was significantly higher in VCM compared with the other subtypes. Expectedly, the expression of *SCN5A*, encoding the cardiac Na^+^ channel, Na_V_1.5, was significantly higher in ACM and VCM compared with SANCM. Immunostaining confirmed these expression patterns in the individual subtype populations (Figure 1(g); Supplemental Figure S1). Robust expression of SHOX2 was observed in SANCM although some expression was seen in ACM as well, consistent with qPCR data. Both SANCM and ACM displayed NR2F2 expression. The expression of MYL2 was almost exclusively found in VCM. Taken together, these results suggest that the directed differentiation protocols yield expected cardiomyocyte subtypes while also alluding to the presence of heterogeneous cell populations, particularly in SANCM and ACM cultures.

### Single cell RNA sequencing of pacemaker and atrial cardiomyocyte cultures

**Figure 2.**
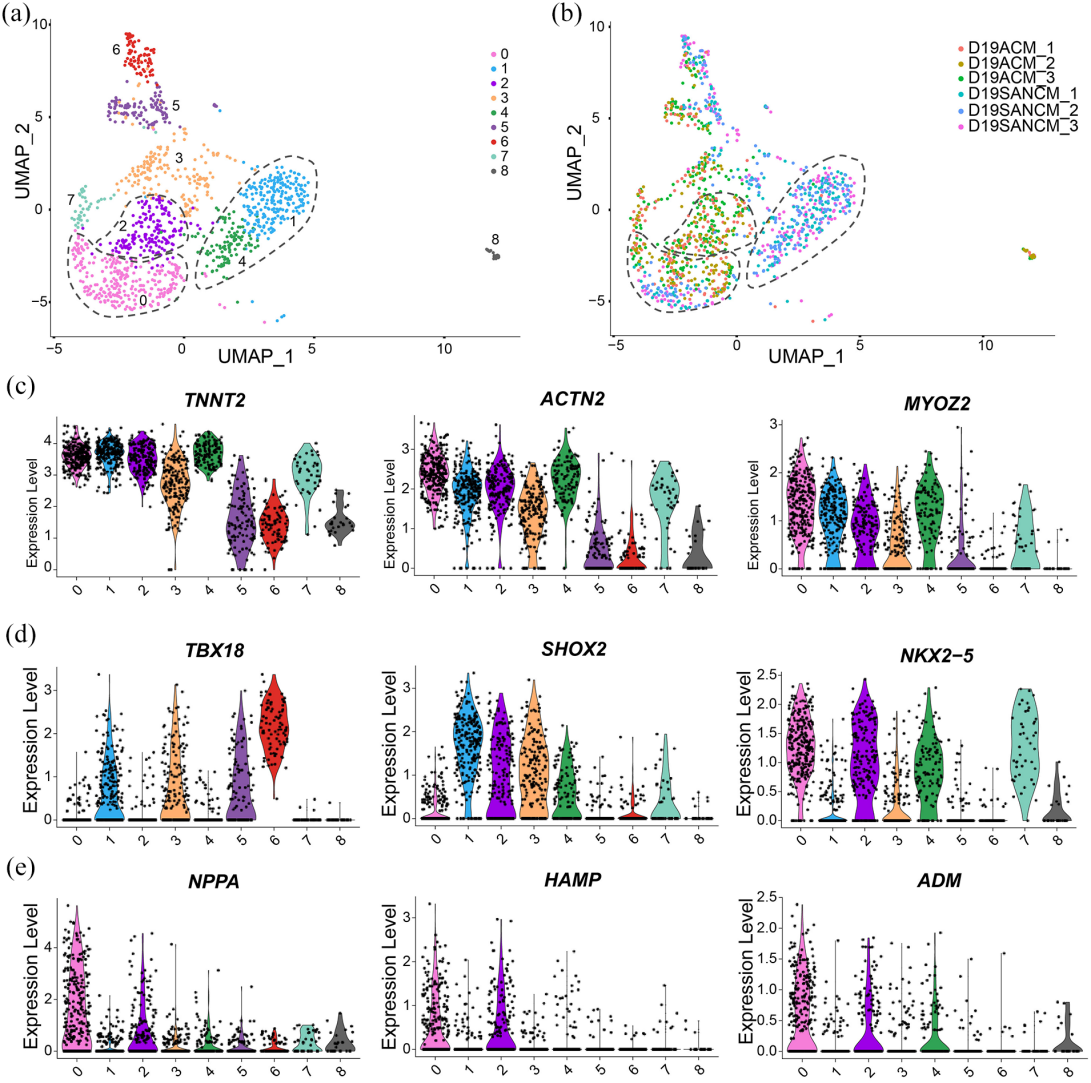
Single cell RNA-sequencing of SANCM and ACM. (a) UMAP representation of single cell transcriptomes of SANCM and ACM at day 20 of differentiation. (b) UMAP showing the original identifier of each cardiomyocyte subtype and plate (1–3). (c–e) Violin plots showing cardiac sarcomeric genes used to identify cardiomyocyte clusters (c), genes used to identify the SANCM populations (d) and genes used to identify ACM populations (e).

To gain further insights into the composition and heterogeneity in SANCM and ACM cultures, we performed single cell RNA sequencing (scRNA-seq) on differentiated populations obtained at day 19 using the SORT-seq protocol.^21^ A total of nine clusters (0–8) were identified in cells resulting from SANCM and ACM differentiations as shown in the UMAP plot in Figure 2(a). In Figure 2(b), we plotted the origin of cells, which demonstrates that clusters 1 and 4 are composed exclusively of cells from SANCM group. Similarly, clusters 2 and 7 are composed of cells from the ACM group alone. In contrast, clusters 0, 3, 5, and 6 contained cells coming from both SANCM and ACM cultures.

Next, we determined the identities of cells using differentially expressed genes in each cluster (Supplemental Figure S2a; Supplemental Excel Table T1). Based on the expression of *TNNT2*, *ACTN2*, and *MYOZ2*, we identified that cardiomyocytes are present in clusters 0, 1, 2, 3, 4, and 7 (Figure 2(c)). The non-cardiomyocyte clusters were identified as smooth muscle cells in cluster 5 (*TAGLN*+, *TMSB4X*+) and pro-epicardial cells in cluster 6 (*ALDH1A2*+, *WT*1+) based on their gene expression signatures (Supplemental Figure S2b and S2c). The identity of cluster 8 could not be discerned.

Importantly, clusters 1 and 4 contained subpopulations of pacemaker cells (Figure 2(d)) corresponding to SAN-head (*TBX18+*, *SHOX2*+, *NKX2-5-*) and SAN-tail regions (*TBX18−*, *SHOX2*+, *NKX2-5+*) as extensively characterized and reported in our recent study.^12^ Furthermore, a third subpopulation of cells from SANCM cultures was found in cluster 0, which also contained atrial cells (*NPPA*+, *HAMP*+, *ADM*+) from ACM cultures. However, these atrial-like cells from SANCM cultures in cluster 0 are distinct from atrial cells from ACM cultures in that they express genes such as *MYL2* and *IRX4* typically absent in atrial cardiomyocytes (Supplemental Figure S3a). Moreover, they also share gene expression with pacemaker clusters 1 and 4 (Supplemental Figure S3b), suggesting that these cells are likely transitional cells found in the SAN, which is an intermediate population with characteristics of both pacemaker and chamber cardiomyocytes.^22^ Lastly, cluster 2 appears to contain a distinct group of cells arising from ACM cultures, which expresses *SHOX2* besides atrial markers (Figure 2(d) and (e)). A closer look at the differentially expressed genes in cluster 2 (Supplemental Excel Table T1) revealed that many of the genes are preferentially expressed in human atrial appendage compared with human left ventricle (Supplemental Figure S4) suggesting that these cells are likely a subpopulation of atrial cells. In sum, SANCM and ACM cultures contain distinct populations of cardiomyocytes.

### Single cell electrophysiological characterization of cardiomyocyte subtypes

**Figure 3.**
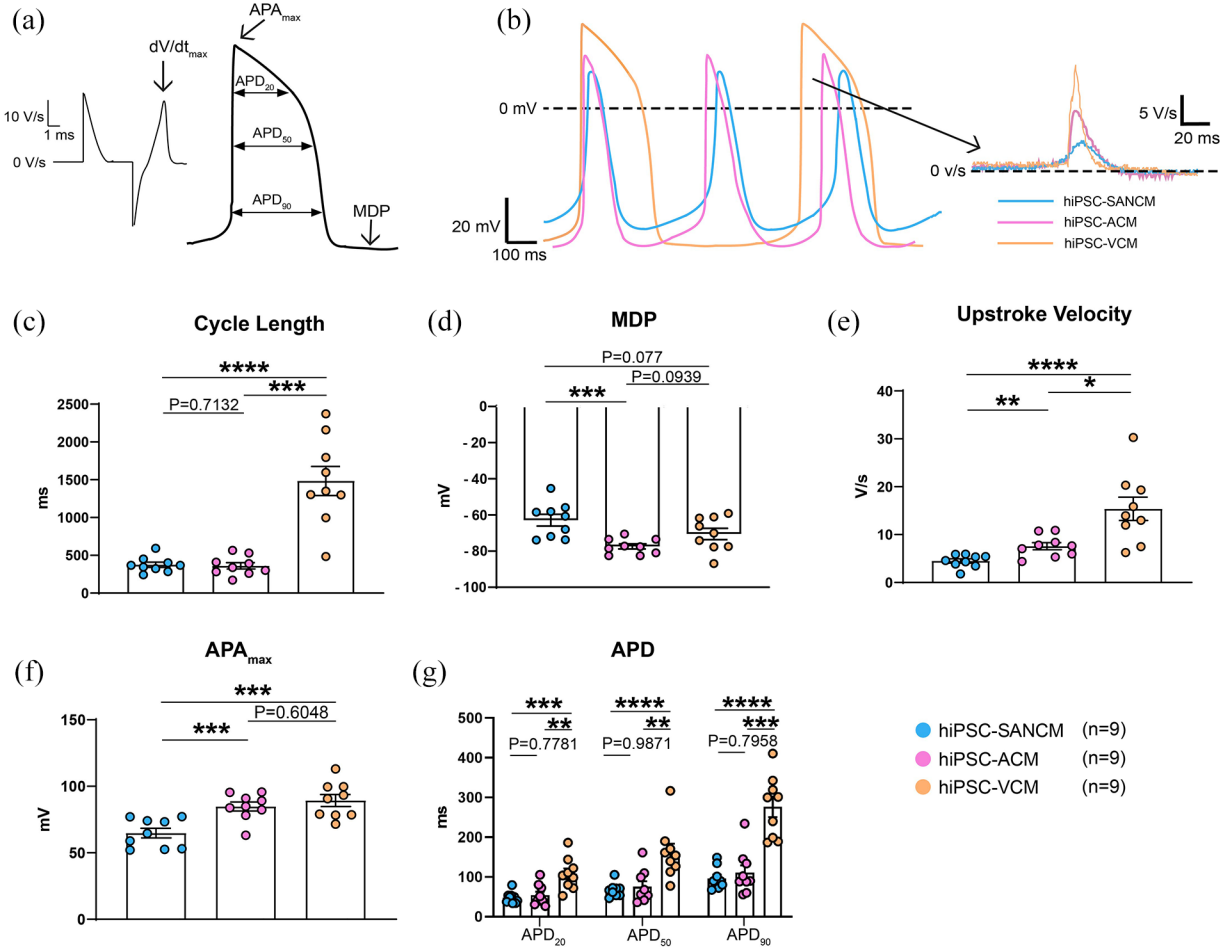
Characterization of cardiomyocyte subtypes by single cell patch-clamp. (a) Illustration of analyzed action potential parameters. (b) Representative spontaneous action potential traces of day 21 SANCM, ACM, and VCM (c–g) cycle length (c) membrane diastolic potential, MDP (d), upstroke velocity, dV/dt_Max_ (e), maximal action potential amplitude, APA_Max_ (f) and action potential duration at 20%, APD_20_, 50%, APD_50_ and 90% repolarization, APD_90_. *N* = 9 cells from four independent differentiations. Error bars, s.e.m. Kruskal Wallis Test followed by Mann Whitney *U*-test for post hoc comparison. *p < 0.05, **p < 0.01, ***p < 0.001, ****p < 0.0001.

To study the electrophysiological properties of all three cardiomyocyte subtypes, we measured action potential (AP) parameters (Figure 3(a)) from single cells dissociated from 2D preparations on day 16 and measured on day 21. Representative traces of spontaneous APs are shown in Figure 3(b), which demonstrate shorter cycle lengths in SANCM (375 ± 34 ms (Average ±SEM); *n* = 9) and ACM (361 ± 43 ms; *n* = 9) compared with VCM (1484 ± 193 ms; *n* = 9) (Figure 3(c)). Furthermore, SANCM displayed a less negative maximal diastolic potential (MDP) (−62.9 mV ± 3.2 mV), slow upstroke velocity (4.5 ± 0.45; see also the inset of Figure 3(b)), small AP amplitude, and short AP durations at 20%, 50%, and 90% of repolarization (APD_20_, APD_50_, and APD_90_, respectively) (Figure 3(d)–(g)). In ACM, MDP was more negative than the other groups (−77.4 ± 1.4 mv) and the APDs were much shorter as in VCM, as reported previously^9^ but similar to SANCM (Figure 3(g)). Lastly, upstroke velocities differed between the groups with the fastest upstroke in VCM (15.4 ± 2.4 V/s). Taken together, these results demonstrate that hiPSC-derived cardiomyocyte subtypes recapitulate salient electrophysiological features of their respective in vivo counterparts.^23,24^

### Effects of Noradrenaline, Carbachol, and Ivabradine on cardiomyocyte subtypes

**Figure 4.**
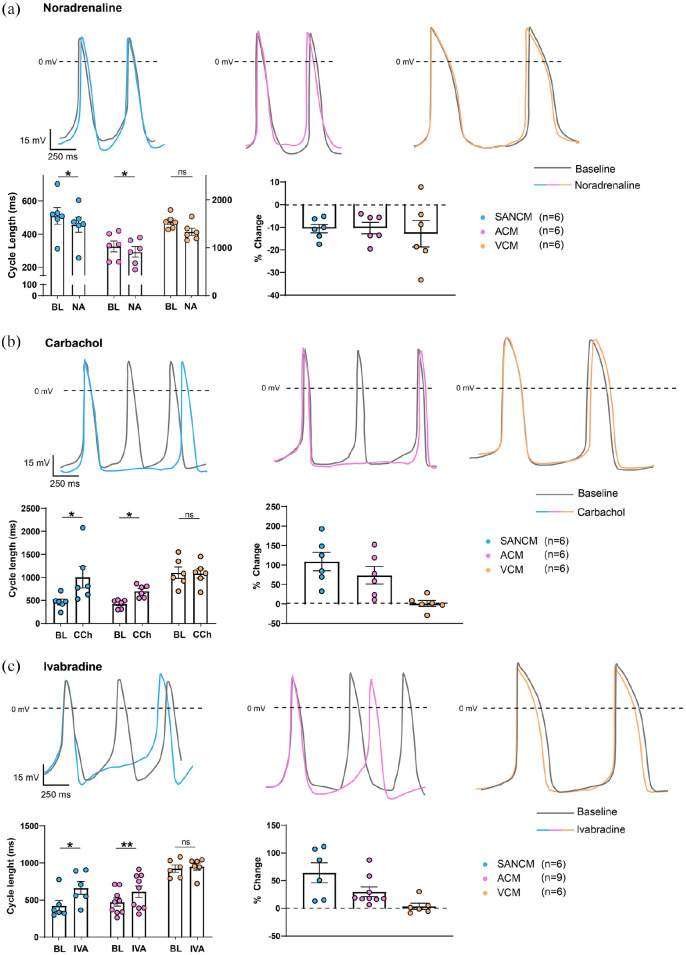
Single cell response to Noradrenaline (NA), Carbachol (CCh) and Ivabradine (IVA). (a) Representative action potential traces of SANCM, ACM and VCM in response to 100 nM NA (top panel). Baseline traces are in gray and individual groups are color coded; Bar graphs demonstrating cycle length in baseline (BL) and after addition of NA (bottom left panel), summarized as % change (bottom right panel). (b) Representative action potential traces of SANCM, ACM, and VCM following addition of 1 µM CCh (top panel); Bar graphs demonstrating cycle length in baseline (BL) and after addition of CCh (bottom left panel), summarized as % change (bottom right panel). (c) Representative action potential traces of SANCM, ACM, and VCM upon treatment with 3 µM Ivabradine (top panel); Bar graphs demonstrating cycle length in baseline (BL) (bottom left panel), summarized as % change (bottom right panel). *N* = 6 from four independent differentiations. Error bars, s.e.m. Wilcoxon signed-rank test, *p < 0.05.

To further characterize the differences between the three cardiomyocyte subtypes, we studied their responses to adrenergic and cholinergic receptor stimulation by noradrenaline and carbachol using patch clamp methodology. In addition, we tested the effects of I_*f*_ blockade by ivabradine. Noradrenaline is an α- and β-adrenergic agonist, which has inotropic and chronotropic effects on cardiomyocytes.^25^ Treatment with 100 nM noradrenaline decreased cycle length in all three groups by about 10% (Figure 4(a)) without changes in any of the action potential parameters (Supplemental Figure S5). Next, we used carbachol to activate I_*KACh*_, which is present in cardiomyocytes of the SAN, atria, and AVN, but not in VCM.^26,27^ 10 μM carbachol prolonged the cycle length in SANCM (108.7 ± 23.7%) and ACM (73.4 ± 22.4%). No changes were observed in any of the action potential parameters (Supplemental Figure S6). Expectedly, carbachol did not influence the cycle length of VCM (0.9 ± 7.7%) (Figure 4(b)), consistent with our previous findings^9,28^ but did cause a small increase in APD_20_ (Supplemental Figure S7). In addition, we investigated the effect of the selective I_*f*_ blocker, Ivabradine^29^ (Figure 4(c)) as I_*f*_ is implicated in generation of diastolic depolarization phase in SAN cells in vivo and in vitro.^30,31^ Following treatment with 3 μM ivabradine, the cycle length in SANCM and ACM was prolonged (64.1 ± 18.1% (SANCM); 29.9 ± 8.6% (ACM), while the effect on VCM was negligible (3.7 ± 5.7%) (Figure 3(c)). Only in ACM, a subtle decrease in upstroke velocity was observed. In VCM however, ivabradine caused a decrease in APD_50_ and APD_90_. These findings further outline the electrophysiological features of hiPSC-derived cardiomyocyte subtypes including the likely presence of I_*f*_ in ACM, albeit at lower magnitude than SANCM.

### Construction and characterization of subtype specific 3D engineered heart tissues

**Figure 5.**
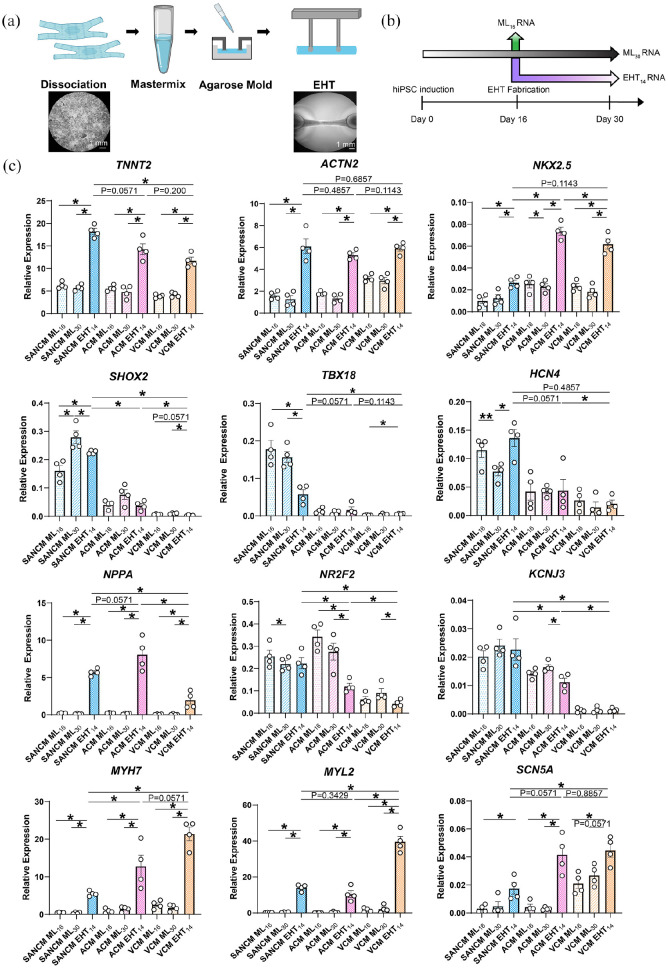
Generation and characterization of subtype-specific engineered heart tissues (EHTs). (a) Schematic representation of EHT fabrication. (b) Timeline of samples taken for qRT-PCR analysis (c) qRT-PCR analysis of gene expression in EHTs. Expression corrected to GEOMEAN of reference genes *RPLP0* and *GUSB. N* = 4 independent differentiations. Error bars, s.e.m. Kruskal Wallis Test followed by Mann Whitney *U*-test for post hoc comparison. *p < 0.05, **p < 0.01.

As single cells do not recapitulate the 3D and multicellular nature of the heart, we generated EHTs at day 16 from all three cardiomyocyte subtypes and measured their gene expression and electrophysiological properties. Figure 5(a) illustrates the process of fabrication of EHTs anchored around flexible silicon posts using fibrin gels as described previously.^32,33^ Spontaneous contractions were observed 5–7 days after casting and tissues could be maintained in culture for at least 30 days. Experiments were performed after 2–3 weeks in culture. Representative videos of EHT subtypes are presented in Supplemental Video V1.

We compared gene expression in EHTs with that of monolayers collected at day 16 (ML_16_) when EHTs were fabricated and to monolayers collected at day 30 (ML_30_) cultured longer to match the 14-day culture period of the EHTs (EHT_14_) (See timeline in Figure 5(b)). The expression of *TNNT2* and *ACNT2* were three-fold higher in all EHT subtypes compared with their monolayer counterparts (Figure 5(c)). The expression of *NKX2-5* was also significantly higher in all EHT subtypes, but the fold change was greater in ACM and VCM EHTs. *SHOX2* expression in day 30 monolayers and EHTs of SANCM was higher than at day 16. Interestingly, the expression of *TBX18* was decreased in SANCM EHTs compared with monolayers. On the contrary, the expression of *HCN4* was higher in SANCM EHTs compared with monolayers at day 30. The expression of *NPPA* was also higher in the EHTs in all groups but the change was greater in ACM and SANCM. *NR2F2* and *KCNJ3* expression was lower in ACM EHTs compared with monolayers. *MYH7, MYL2* expression was also significantly higher in all EHT subtypes but the increase was greater in VCM. The expression of *SCN5A* was significantly higher in all three EHT subtypes with the largest increase in ACM and VCM EHTs. Overall, stretch-responsive genes such as *NPPA*, *MYH7* and other sarcomeric genes appear to have the most pronounced expression changes upon EHT culture.

### Effects of Noradrenaline, Carbachol, and Ivabradine on EHTs

**Figure 6.**
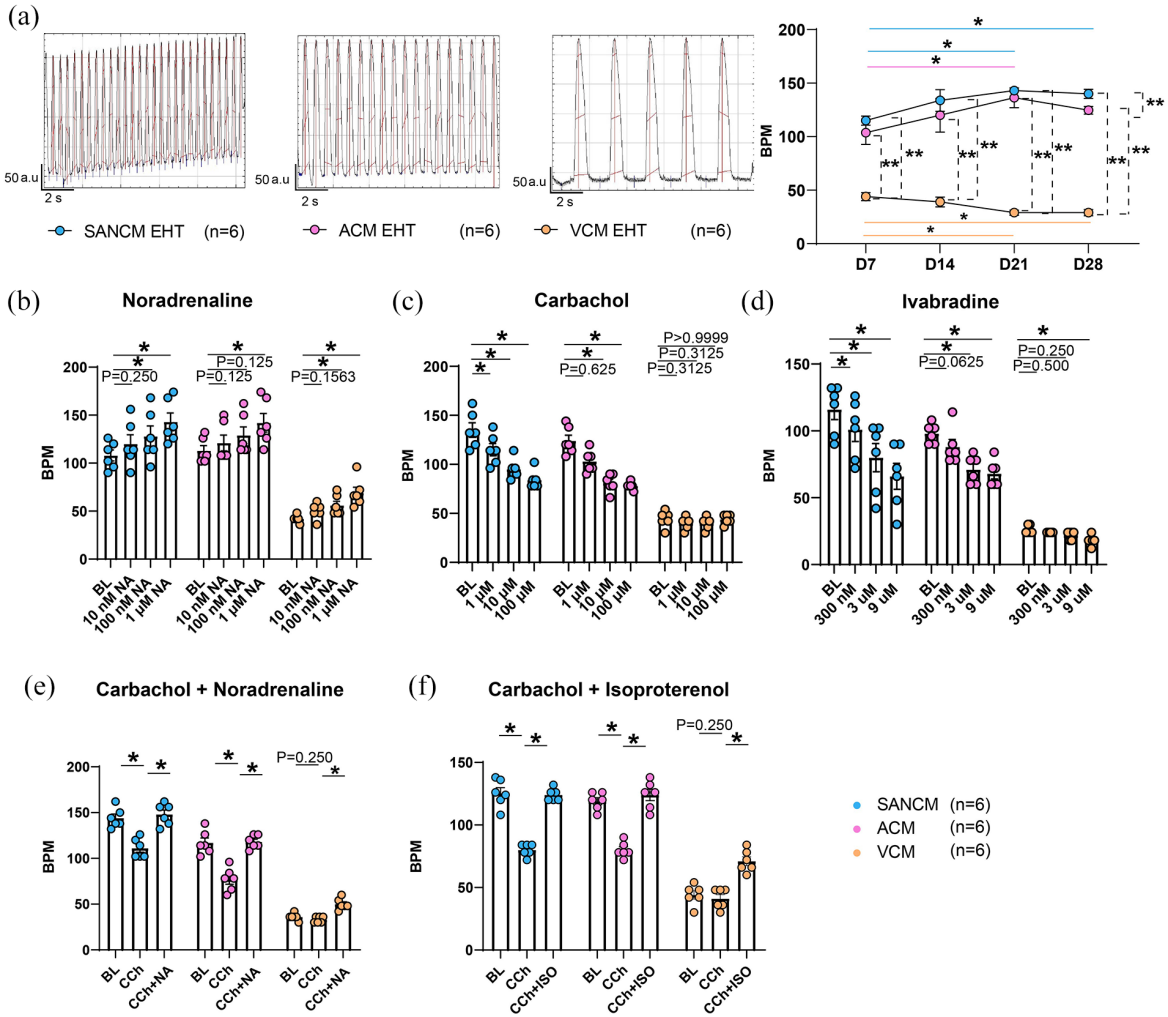
EHT response to Noradrenaline, Carbachol and Ivabradine. (a) Representative beating profiles and summary of beating rates recorded at 7, 14, 21, and 28-days post fabrication in SANCM-, ACM-, and VCM EHTs. (b–d) Beating rate in response to Noradrenaline (b), Carbachol (c) and Ivabradine (d). *N* = 6 EHTs/subtype from four independent differentiations (e) Beating rate in baseline (BL), in response to 100 µM carbachol (CCh) and subsequent treatment with 1 µM Noradrenaline (NA). (f) Beating rate in baseline (BL), in response to 100 µM carbachol (CCh) and subsequent treatment with 10 µM isoproterenol (Iso). *N* = 6 from four independent differentiations. Error bars, s.e.m. Pairwise error calculated with Wilcoxon’s Test. Multiple group comparison with Kruskal Wallis Test followed by Mann Whitney *U*-test for post hoc comparison, *p < 0.05.

We determined beating rate per minute (BPM) in EHTs over a period of 28 days. Similar to single cells, SANCM (140 ± 4.3 BPM) and ACM (124 ± 3.6 BPM) EHTs displayed faster beating rates compared with VCM (29 ± 2.9 BPM) (Figure 6(a)). Next, we measured the response of 21-day old EHTs to the same drugs tested on single cells in Figure 4 to compare the effects. In response to noradrenaline, all three EHT subtypes demonstrated faster beating rates, which was significant in SANCM and VCM at 100 nM and all three tissue subtypes at 1 uM (108 ± 6 to 143 ± 9 BPM in SANCM at baseline and 1 µM respectively; 113 ± 5to 142 ± 10 in ACM; 42 ± 2 to 69 ± 6 BPM in VCM) (Figure 6(b)). On the other hand, carbachol had a dose dependent decrease on beating rate in SANCM (135 ± 7 to 84 ± 4 BPM at baseline and 100 µM respectively) and ACM (124 ± 6 to 78 ± 2 BPM) EHTs, but not in VCM EHTs (Figure 6(c)). Similarly, ivabradine decreased beating rates of SANCM (116 ± 7.5 to 66 ± 9.7 BPM at baseline and 9 µM, respectively) and ACM EHTs (98 ± 3.0 to 68 ± 4.0 BPM) illustrating an important role for I_*f*_ in the spontaneous activity of these cell types (Figure 6(d)). Whilst lower concentrations of ivabradine had no effect on VCM EHTs, the highest concentration tested (9 µM), caused a 30% decrease in beating rate (26 ± 1.3 to 18 ± 1.5 bpm), likely due to aspecific effects on the delayed rectifier K^+^ current resulting in delayed repolarization.^34^ Interestingly, no significant differences in contractile properties were observed between subtype EHTs at baseline or in response to noradrenaline and carbachol. However, ivabradine had a negative inotropic effect in SANCM and VCM (Supplemental Figure S8).

Lastly, we evaluated the feasibility of using subtype specific EHTs for simultaneous testing of rate modulating drugs. Application of 100 µM carbachol slowed the beating rate in SANCM and ACM EHTs by approximately 30%. Subsequent stimulation of the beta-adrenergic receptors, by either 1 µM noradrenaline or 10 µM Isoproterenol, restored beating rates to basal levels in both groups (Figure 6(e) and (f)), similar to our observations in rabbit SAN cells.^25,35^ Carbachol had no effect on VCM EHTs while treatment with noradrenaline or isoproterenol resulted in a 52% increase in beating rate (Figure 6(e) and (f)). Altogether, the responses of drugs tested in EHTs were similar to those observed in single cells.

### Assessment of conduction velocities in monolayers and EHTs

**Figure 7.**
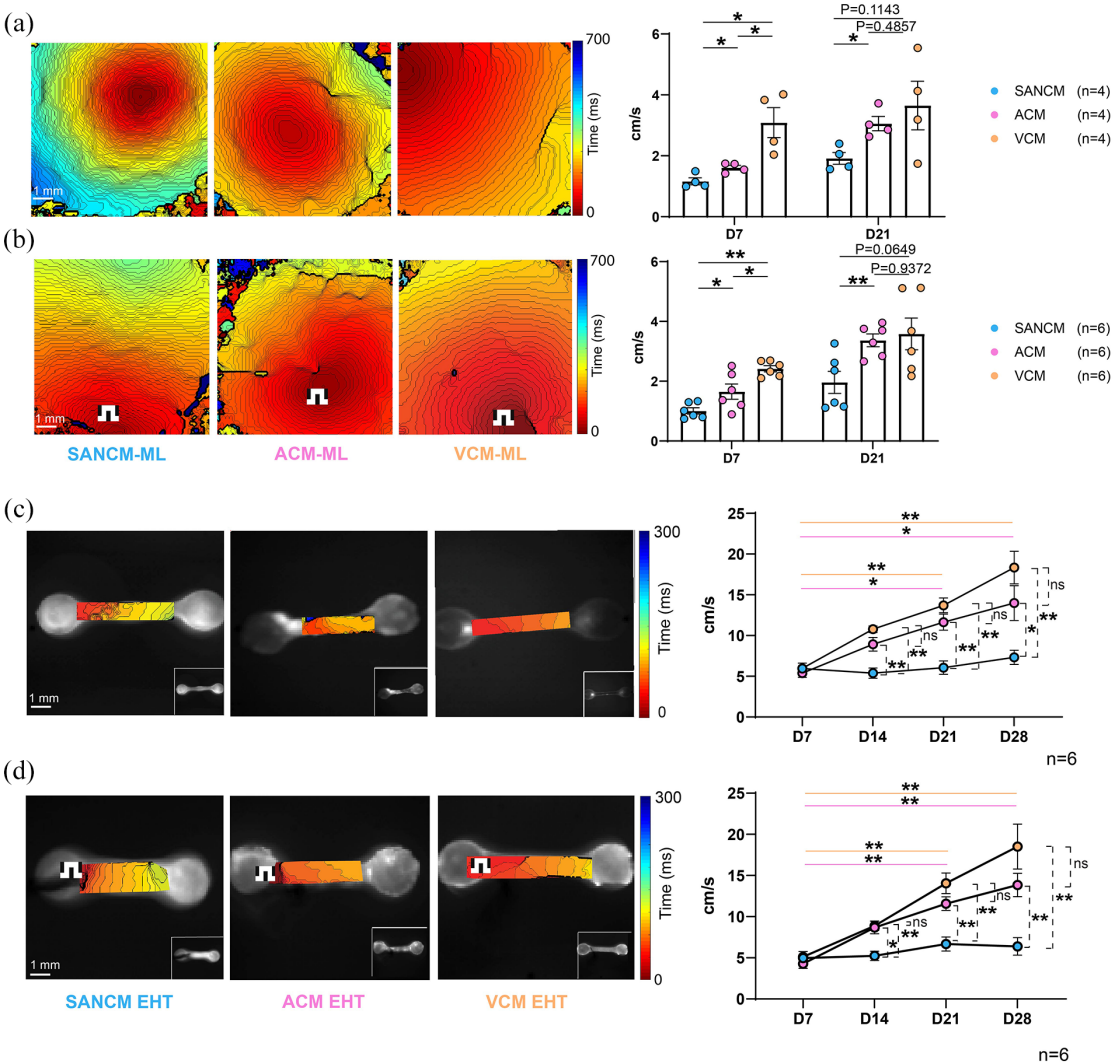
Impulse propagation in monolayers and EHTs. (a and b) Representative activation maps (left. panels) and summarized bar graphs (right panels) of spontaneous (a) and paced at 1 Hz (b) monolayers at 7 and 21 days post seeding. (c and d) Representative activation maps (left panels) and summarized data (right panels) of spontaneous (c) and paced (d) engineered heart tissues on 7-, 14-, 21-, and 28-days post fabrication. Error bars s.e.m. *N* = 4–6 from four independent differentiations. Multiple group comparison with Kruskal Wallis Test followed by Mann Whitney *U*-test for post hoc comparison, *p < 0.05, **p < 0.01 Mann Whitney Test. *p < 0.05, **p < 0.01 versus indicated sample.

To assess the speed of impulse propagation (conduction velocity) in EHT subtypes and their monolayer counterparts, we performed optical mapping. Conduction velocity in SANCM (1.162 ± 0.115 cm/s) monolayers was slower than in ACM (1.61 ± 0.07 cm/s) and VCM (3.09 ± 0.49 cm/s) 7 days post seeding (Figure 7(a)). After two more weeks of culture (21 days post seeding), there was only a small increase in conduction velocities in all three groups (1.92 ± 0.19 cm/s SANCM; 3.06 ± 0.24 cm/s ACM; 3.65 ± 0.8 cm/s VCM). Nonetheless, conduction velocities in SANCM were still slower compared with ACM and VCM (Figure 7(a)). Similar observations were obtained when monolayers were paced at 1.0 Hz (Figure 7(b)). In EHTs, measurements were acquired over a period of 28 days. At day 7 post fabrication, all three subtypes had similar conduction velocities of around 5 cm/s (5.94 ± 0.44 cm/s SANCM; 5.39 ± 0.53 cm/s ACM; 5.96 ± 0.65 cm/s VCM. However, by day 14, conduction velocities in ACM (8.92 ± 0.83 cm/s) and VCM (10.78 ± 0.41 cm/s) increased 1.5–2-fold (Figure 7(c)) while conduction velocity in SANCM remained around 5 cm/s (5.39 ± 0.63 cm/s). Thereafter, ACM and VCM EHTs displayed a steady increase in conduction velocity over time to a maximum of 13.99 ± 2.14 cm/s and 18.35 ± 2.00 cm/s, respectively, on day 28 indicating a positive effect of 3D culturing on these cell types (Figure 7(c)). Pacing the tissues at 1.5 Hz resulted in similar findings in all three groups (Figure 7(d)). These findings demonstrate that the conduction velocity differences between the subtypes were more pronounced in the EHTs than in the monolayers suggesting the utility of the 3D models for comparative assessments.

### A rudimentary in vitro model of the pacemaker-atrial interface

**Figure 8.**
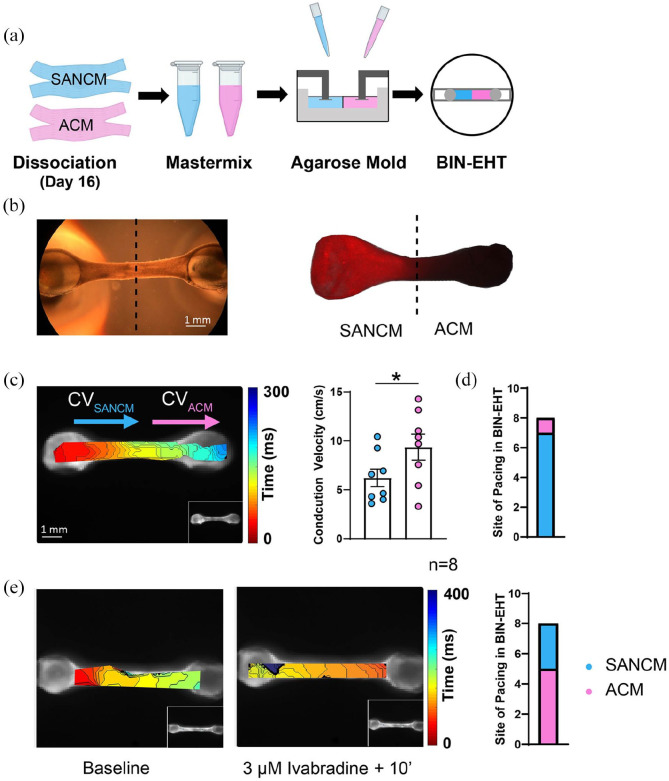
Fabrication of binary engineered heart tissues (BIN-EHTs). (a) Schematic illustrating the method to combine hiPSC-SANCM (cyan) and hiPSC-atrial cells (magenta) in BIN-EHTs. (b) Bright field and fluorescent images of a heteropolar EHT, where SANCM were labeled with DiI prior to fabrication. (c) Representative activation map (left) and summarized data (right) showing conduction velocities in SANCM, and ACM parts of BIN-EHTs. (d) Bar graph depicting site of impulse initiation in 8 BIN-EHTs. (e) Isochronal maps (left) demonstrating impulse initiation under baseline (BL) and 10 min after treatment with 3 µM ivabradine in a representative EHT. Summarized data from 8 BIN-EHTs depicts site of impulse initiation. Error bars s.e.m. *N* = 8 from four independent differentiations. Wilcoxon’s Test, *p < 0.05.

In order to assess the behavior of SANCM when co-cultured with ACM, we generated simplistic binary tissues (BIN-EHT) that combined the two cell types. Prior to casting, two hydrogel mixes, each containing either SANCM or ACM were prepared. These cell-hydrogel mixtures were subsequently pipetted onto opposite ends of the agarose mold, which proceeded to gently combine at the center forming one unified tissue (Figure 8(a)). Using DiI labeled SANCM fraction, we verified the heteropolar localization of the two cell types in the EHT (Figure 8(b)).

To evaluate impulse propagation in BIN-EHTs, we carried out conduction velocity measurements 14 days post fabrication. A representative isochronal map is shown in Figure 8(c). On the SANCM end, conduction velocities were slower (6.22 ± 0.90 cm/s) compared with the ACM end (9.36 ± 1.32 cm/s) (Figure 8(c)). This is comparable to the conduction velocities observed in individual SANCM (5.24 ± 0.56 cm/s) and ACM (8.75 ± 0.92 cm/s) EHTs at day 14. Importantly, in the BIN-EHTs, SANCM end was the leading pacemaker in seven out of eight tissues (Figure 8(d)). We then evaluated the effect of ivabradine on BIN-EHTs. Following superfusion with 3 μM ivabradine, the beating rate of the tissues markedly slowed down and in five out of eight tissues, lead pacing site shifted from SANCM to the ACM side of the EHT (Figure 8(e)). All in all, these data suggest that dual EHT constructs composed of SANCM and ACM are physiologically relevant in vitro models for studying impulse formation and propagation.

## Discussion

A thorough characterization of hiPSC-derived cardiomyocytes will aid the development of advanced, physiologically relevant models suitable for drug discovery and regenerative medicine. Here, we rigorously characterized the molecular and electrophysiological features of three major hiPSC-derived cardiomyocyte subtype models. SANCM, ACM and VCM were generated using directed differentiation protocols as reported previously.^9,12^ Inhibition of WNT signaling at the cardiac mesoderm stage is a widely used strategy to generate cardiomyocytes.^36,37^ Differences in the methods used for mesoderm induction, that is, growth factor or small molecule strategies or genetic and epigenetic differences between cell lines may influence the identity of the resulting cell types. In our cell line, mesoderm induction using Activin-A, BMP4 and WNT activator, CHIR99021 and subsequent inhibition of WNT signaling using XAV939 resulted in cultures containing ventricular and outflow tract cells as shown recently.^12^ For differentiation toward atrial lineage, we and others have previously shown a role for the activation of RA signaling.^9,11,38^ In the current study, we used a RA receptor agonist, BMS 753 in lieu of RA. Given that RA is a highly unstable, photo- and thermosensitive compound, we tested alternatives and identified that BMS 753 is equally effective for atrial differentiation. For differentiation of pacemaker cells of the SAN, we optimized the application of BMP and RA signaling based on a method demonstrated before.^10^

Gene expression analysis on bulk populations of the three cardiomyocyte subtypes identified that the expression of *TNNT2* was higher in SANCM, and ACM as opposed to higher expression of *ACTN2* in VCM. In a recent study, we showed that our VCM cultures contain a subpopulation of embryonic outflow tract cardiomyocytes (*ISL1*+, *PITX2*+, *HAPLN1*+), which express lower *TNNT2* compared with ventricular and pacemaker cardiomyocytes.^12^ On the contrary, ventricular cardiomyocytes in VCM cultures express more *ACTN2* compared with pacemaker and outflow tract cardiomyocytes, which explains the differential expression of sarcomeric genes in the bulk populations analyzed in the current study. Furthermore, qPCR and immunocytochemistry of subtype-associated markers suggested the presence of ACM in SANCM and SANCM in ACM cultures. We clarified this observation through scRNA-seq, which demonstrated that atrial genes (*NKX2-5; NPPA*) detected in SANCM arise from subpopulations of pacemaker cells such as SAN-tail and SAN-transitional cells that also express these genes. Similarly, the expression of *SHOX2* in the ACM cultures was tracked to a subpopulation of cells that expressed this gene while also expressing other atrial markers. In the embryonic heart, *SHOX2* is expressed in the sinus venosus and its progenitors, which contribute to both the atria and the SAN.^39,40^ Heterogeneous populations of atrial cells have also been reported in the adult human heart strengthening the possibility of subpopulations with different developmental origins.^41^ Our findings emphasize the importance of meticulous characterization of cells obtained from directed differentiation protocols in each lab as individual culture protocols as well as genetic and epigenetic variations in cell lines tend to influence the outcomes. Awareness of the composition of resulting cardiomyocyte cultures and identifying methods to purify desired cardiac subtypes is essential for effective application of these cells for preclinical purposes such as pharmacological screenings as well clinical purposes such as cell therapy.

Electrophysiological characterization provided further insight into the differences between the three cardiomyocyte subtypes. A more positive MDP, prominent diastolic depolarization, lower AP amplitude, and slower AP upstroke velocity distinguished SANCM from the other subtypes, in line with AP properties of isolated human SAN cells.^24^ Detailed patch-clamp and Ca^2+^ homeostasis experiments are required to address these differences (which is beyond the scope of the current study) but may be related to a higher expression of *HCN4* (Figure 1(d)) and larger depolarizing I_*f*_ current, resulting in less negative MDP. AP amplitude, and AP duration may be changed consequently. In addition, the APD was shorter in ACM compared to VCM, likely due to the presence of a large repolarizing ultrarapid delayed rectifier K^+^ current.^9,42^

We also evaluated the sensitivity of the various cardiomyocyte subtypes to agents that modulate heart rate. 100 nM noradrenaline elicited a positive chronotropic response in all groups, although the effect did not reach significance in VCM. Noradrenaline also failed to elicit any changes to action potential parameters such as APD. This is likely a result of a combination of factors but primarily APD prolongation driven by an increase in I_Ca,L_ which is counteracted by an increase in repolarizing slow delayed rectifier current I_Ks_^43^ and shortening of action potential duration at faster beating rates.^44^ In response to 1 µM carbachol, a negative chronotropic effect was observed in SANCM and ACM. Carbachol had no effect on VCM in line with in vivo findings that I_*KACh*_ is restricted to the SAN, atria and AVN^26,27^ and previous findings in hiPSC-derived cardiomyocytes.^9,28^ Carbachol treatment did not affect APD consistent with previous findings *in vivo* as well as in hiPSC-CMs^28^ possibly due to a decrease in delayed rectifier potassium currents, which counteracts carbachol induced inhibition of I_Ca,L_ resulting in almost no change in APD.^45,46^ Spontaneous activity seen in immature hiPSC-derived cardiomyocytes has been attributed at least in part to the presence of I_*f*_,^47–49^ which is an important determinant of automaticity in the SAN.^30^ 3 µM ivabradine evoked a negative chronotropic response only in SANCM, and in ACM albeit to a lower magnitude suggesting functional presence of I_*f*_ in these cell types. In human fetal stages corresponding to the first trimester, *HCN4*, the predominant isoform for I_*f*_ in the human SAN is also expressed in the developing atria^50^ and the presence of I_*f*_ in hiPSC-derived ACM thus suggests their relative immaturity. In our study, VCM did not respond to ivabradine in contrast to previous observations,^47–49^ which likely contained a mix of different cardiomyocyte subtypes. The effect on APD seen in ivabradine treated VCM can be explained by the blockade of I_Kr_ leading to an overall reduction in available repolarizing current causing prolongation of APD.^51^ This does not occur in ACM and SANCM as they harbor less intrinsic I_Kr_.

Next, we asked whether 3D culturing in EHTs would influence the properties of the cardiomyocyte subtypes. EHTs fabricated in a strip format have been shown to promote certain aspects of maturation such as sarcomere alignment and improved force of contraction.^32^ One difference with previous reports that also evaluated EHTs^6,7,52^ is that comparisons in those studies were restricted to chamber subtypes, that is, ACM and VCM. Moreover, differentiation protocols employed for cardiomyocyte generation were based on embryoid body-based cultures in contrast to monolayer-based methods used in our study.

Gene expression analysis of respective subtype monolayers and corresponding EHTs revealed that 3D culturing in EHTs noticeably impacts stretch-associated genes. Consistent with a previous study, we did not observe any remarkable changes in ion channel genes from 2D to 3D cultures with the exception of *SCN5A*.^32^ We also assessed whether the chronotropic agents tested in single cells would elicit the same response in EHTs. Noradrenaline elicited a positive chronotropic effect in all EHT subtypes in line with previous studies in vivo and in vitro^53,54^ but no inotropic response was observed. Early studies that assessed the effect of noradrenaline reported a positive inotropic effect in vivo.^55-57^ However, clinically, noradrenaline is used as a vasopressor rather than as an inotrope due its less potent effects compared with isoproterenol or dobutamine.^58^ In hiPSC-CMs in vitro, noradrenaline has been shown to have a positive inotropic effect albeit large differences in the magnitude of the effect (effect on single cells, 300% increase in contraction force^54^; effect on EHTs, 10% increase in contraction force^49^). In our study, we did not observe an inotropic effect of noradrenaline on any of the subtypes, which may also be a result of suboptimal expression of alpha and/or beta-adrenergic receptors in the hiPSC line we studied, as reported in a prior study outline one of the limitations of this model system.^59^ The negative chronotropic effect of 100 µM carbachol on ACM-EHTs and SANCM-EHTs are in line with previous studies in mammalian cardiomyocytes,^25,60^ confirming the presence of functional I_KACh_ in these subtypes. Furthermore, carbachol caused a small, but non-significant negative inotropic effect at the highest concentration tested (100 µM) in ACM and VCM EHTs. It has been reported that muscarinic receptor activation affects force independent of I_KACh_ activation, and likely the negative inotropic effect observed is due to an effect on calcium current in turn leading to reduction of contraction force.^61,62^ 3 µM Ivabradine evoked a negative chronotropic effect in SANCM-EHTs and ACM-EHTs, analogous to the effect observed in single cells. At 9 µM, the highest concentration tested, the beating rate of VCM-EHTs also slowed down likely due to non-specific effects of the drug at higher concentrations.^51^ In addition, 9 µM Ivabradine caused a negative inotropic effect in SANCM- and VCM-EHTs but interestingly, not in ACM. The effect of ivabradine on cardiac contractility remains unclear as previous studies have reported varying and inconsistent effects.^63,64^

There were clear differences in conduction velocities between the three EHT subtypes. ACM and VCM showed a steady increase over the 28-day window, SANCM maintained relatively slower conduction velocities. These findings are in line with observation in vivo where conduction velocity is markedly slower in human SAN (5 cm/s) and AVN (5 cm/s) compared with the atrial and ventricular myocardium (50–80 cm/s).^37,38^ Notably, there was a three-to-four-fold increase in conduction velocity of ACM and VCM EHTs in comparison with the monolayer counterparts after 21 days of culture likely due to an increase in I_Na_ inferred from increase in *SCN5A* expression. The peak conduction velocity values of 13 cm/s seen in our ACM EHTs is markedly higher than 4 cm/sec reported in ring-shaped EHTs.^7^ Other previous studies that assessed deflectable pole-based ACM EHTs^6,52^ did not assess conduction velocity. On the other hand, average conduction velocity of 18 cm/s in VCM EHTs are comparable to previous studies.^7,65,66^ However, they are at least three-fold lower than conduction velocities reported for adult atrial and ventricular tissue in vivo.^67,68^ Conduction velocities observed in SANCM are largely similar to the in vivo situation.

Finally, we modeled the SAN-atrial interface using simple heteropolar EHTs composed of the two cell types. In the heart, the source-sink balance between the SAN and atria is essential for setting the pacemaker rate and for activation of the right atrial cardiomyocytes. Our results in BIN-EHTs indicate that under baseline conditions, SANCM maintain the lead. However, upon treatment with ivabradine, ACM take over in a majority of the tissues. Since any I_*f*_ dependent pacemaking mechanism would also be blocked by ivabradine, it appears likely that a calcium-clock dependent mechanism underlies this switch in impulse formation to ACM. This is a limitation due to immaturity of hiPSC-derived cardiomyocytes. Nevertheless, the SAN-atrial interface model is valuable to study mechanisms related to impulse propagation in pacemaker cells in health as well as disease (e.g. SAN exit block). It is interesting to observe that despite similar cycle length in SANCM and ACM (because of their immaturity), SANCM take the lead in BIN-EHTs, suggesting that this model is ideal for evaluating which properties/features give this unique advantage to pacemaker cells, thus allowing to uncover the minimal structural and electrical features necessary for a functional pacemaker-working myocardium interface. These advances are expected to advance the creation of next-generation in vitro models for disease modeling and cell-based biological pacemakers for regenerative therapies.^69,70^

## Methods

### Maintenance of hiPSCs and differentiation to cardiomyocyte subtypes

hiPSC line LUMC0099iCTRL#04 was maintained in mTESR1 medium (Stem Cell Technologies, #5850) on growth factor reduced Matrigel, (Corning #356234) at 37°C/5% CO_2_. For cardiomyocyte differentiation, cells were seeded at a density of 10 × 10^3^ cells per cm^2^. Differentiation was induced when hiPSCs reached 80%–90% confluency. BPEL medium^71^ supplemented with 20 ng/mL Activin-A (Miltenyi Biotec #130-115-012), 20 ng/mL BMP4 (R&D systems, #314-BP-101/CF) and 1.5 μM CHIR99021 (Axon Medchem, #1386) was used to initiate differentiation to cardiac mesoderm. Three days after initiation, medium was replaced with BPEL containing 5 μM XAV939 (Bioscience, #3748/10) for ventricular differentiation, in combination with BMS 753 (Tocris # 3505) for atrial differentiation or in combination with 2.5 ng/mL BMP4 (R&D Systems 314-BP-010/CF), 5 μM SB431542 (Tocris #1614), 250 nM RA and 250 nM PD173074 for sinoatrial nodal differentiation. Differentiation medium was replaced with BPEL after 48–72 h, and cells were refreshed every 3 days thereafter.

### RNA isolation and quantitative Real-Time PCR (qRT-PCR)

Total RNA was isolated using RNAzol RT (Sigma-Aldrich, #R4533) according to the manufacturer’s protocol with an additional chloroform-based phase separation step. Briefly, cells or tissues were lysed with 350 μL RNAzol RT followed by the addition of 140 μL RNase-free water and samples were centrifuged for 15′ at 12,000 × *g* at room temperature. Supernatant was transferred to a clean tube and 4-Bromoanisole (Aldrich, #B56601-100 G) equivalent to 0.5% of the supernatant volume was added to the supernatant. Samples were centrifuged for 10′ at 12,000 × *g* at RT. Supernatant was transferred to a clean tube, 70 μL of chloroform (Merk, #67-66-3) was added and samples were centrifuged for 15′ at 10,000 × *g* at 4°C. An equal volume of 100% Isopropanol (Merk, #24137-1 L-M) was added to the supernatant and centrifuged for 10′ at 12,000 × *g* at 4°C. RNA pellet was washed 2x with 75% Ethanol (Merk, #64-17-5). RNA was reconstituted in 30 μL RNAse free water.

For cDNA synthesis, 1 µg RNA was treated with DNAseI (Thermofisher #18068015), and reverse transcribed using Superscript II reverse transcriptase (Invitrogen #18068014), and oligo-DT primers according to the manufacturer’s protocol. 10 ng was used as input for each qPCR reaction. qPCR primers are listed in Supplemental Table S1. Signal detection was performed using LightCycler 480 SYBR Green master 1 (Roche; 04887352001). The amplification protocol was as follows: 5 min 95°C followed by 45 cycles of 10 s 95°C, 20 s 60°C, and 20 s 72°C. Data was analyzed using LinRegPCR.^72^ For data normalization, *RPLP0* & *GUSB* were used as reference genes.

### Immunocytochemistry

Cardiomyocytes were seeded on glass coverslips and fixed with 4% paraformaldehyde. Cells were permeabilized with 0.1% Triton-X (Sigma T8787), and a blocking step was performed with 4% swine serum (Jackson Immunoresearch, #014-000-121) for 1 h at room temperature. Primary and secondary antibodies were diluted in 4% swine serum and incubated at room temperature for 1 h. Nuclei were stained with DAPI. Imaging was performed with the LEICA TPS SP8X. Image visualization and processing was performed with LAS-X (Leica) software. The following primary antibodies and dilutions were used TNNT2, 1:1000 (Abcam, #ab45932); ACTN2, 1:800 (Sigma Aldrich, #A7811), SHOX2, 1:200 (Abcam, #ab55740), NR2F2, 1:200 (R&D Systems, #PP-H7147-00), MYL2, 1:200 (Abcam, #ab79935).

### Flow cytometry

Cardiomyocytes were dissociated with 1x TrypLE Select (ThermoFisher #12563011) for 15–20 min at 37°C. For cell staining using antibody against TNNT2 (Miltenyi Biotec, #130-118-354) or isotype control (Miltenyi Biotec #130–118-354), cells were fixed and stained using commercially available fixation buffer (Biolegend #420801) and intracellular staining buffer (Biolegend #421002) according to manufacturer’s instructions. Antibody incubations were performed for 30 min on ice in the dark. Acquisition was performed with FacsCanto II Cell Analyzer (BD Biosciences). Data was analyzed using FLowJo v10.

### Single Cell RNA sequencing

Single cell sequencing was performed from two independent differentiations using SORT-seq method.^21^ Preparation of single-cell libraries was performed using the CEL-Seq2 protocol.^21,73^ Paired-end sequencing was performed on the NextSeq500 platform using 1 × 75 bp read length kit.

Reference genome annotation and bioinformatics analysis were performed as described recently^12^ using the R toolkit Seurat version 4.^74^ Dimensionality reduction was performed using the top 20 principal components and seed set to 2020. Original sequencing data has been deposited in NCBI GEO repository under the accession number GSE189782.

### Single cell patch-clamp

Day 16 cardiomyocytes were dissociated using 1x TryPLE Select and plated on Matrigel coated coverslips at a density of 7.0 × 10^3^ per coverslip. After 1 week, cells with a smooth surface and intact membrane were chosen for measurements. APs were recorded at 37°C with the amphotericin-B-perforated patch-clamp technique using Axopatch 200B Clamp amplifier (Molecular Devices Corporation). Measurements were carried out in Tyrode’s solution containing (in mM): 140 NaCl, 5.4 KCl, 1.8 CaCl_2_, 1.0 MgCl_2_, 5.5 glucose and 5.0 HEPES. pH was adjusted to 7.4 with NaOH. Pipettes (borosilicate glass; resistance 1.5–2.5 MΩ) were filled with a solution containing (in mM): 125 K-gluconate, 20 KCl, 10 NaCl, 0.44 mM amphotericin-B, and 10 mM HEPES. pH was adjusted to 7.2 with KOH. Signals were low-pass-filtered (cut off frequency 10 kHz) and digitized at 40 kHz. Membrane potentials were corrected for the estimated change in liquid junction potential (Barry & Lynch, 1991). Data acquisition and analysis were performed using custom software.

### Engineered heart tissue fabrication

Fibrin-based EHTs were generated as described previously.^33^ Each EHT contained 1.5–2.0 × 10^6^ hiPSC-derived cardiomyocytes, differentiated for 16 days. Briefly, casting molds were generated with Teflon spacers (EHT technologies #C0002) placed in 2% liquid agarose molds (Millipore #121853) in 24-well plates. Agarose was allowed to solidify for 15 min and silicone racks containing two posts per well (EHT Technologies; C0001) were positioned in the casting molds. The cell-hydrogel suspension consisting of cardiomyocytes, fibrinogen, thrombin and Matrigel was then poured around the posts and incubated at 37°C/5% CO_2_ for 1.5–2 h to allow polymerization. EHTs adhered to the silicon racks and were transferred to culture medium containing DMEM/F12 low glucose (Sigma Aldrich D5546), 5% heat inactivated horse serum (ThermoFisher #26050088); 1% penicillin/streptomycin (ThermoFisher #15070-063); 0.1% (w/v) Aprotinin (Sigma Aldrich A1153) and 0.1% Insulin (Sigma Aldrich I9278). Medium was replenished thrice a week. EHTs demonstrated regular contractions 5–7 days after fabrication.

### Contraction analysis

Contraction analysis on EHTs was performed using MUSCLEMOTION.^75^ Briefly, 10-s videos were captured and converted to raw .avi files for analysis and then imported into Fiji using the MUSCLEMOTION software macro, which computes the contraction parameters.

### Optical mapping

Cardiomyocytes were incubated in media containing 20 µM Di-4 Anneps for cell monolayers and in combination with 10 µM Blebbistatin for EHTs at 37°C for 25 min. EHTs and monolayers were placed in a water bath containing a modified Tyrode’s solution containing (in mM) NaCl 140, KCl 5.4, CaCl2 1.8, MgCl_2_ 1.0, glucose 5.5 maintained at pH 7.4 by equilibration with a mixture of 95% O_2_ and 5% CO_2_. Excitation light was provided by a 5 W power LED (filtered 510 ± 20 nm). Fluorescence (filtered>610 nm) was transmitted through a tandem lens system on CMOS sensor (100 × 100 elements, sampling rate 5 kHz, MICAM Ultima). Optical action potentials were analyzed using custom made software.^76^ Pacing of EHTs and monolayers was provided by a twin tipped micro electrode (900 μA, 2 ms pulse) and custom-made software.

### Statistical analysis

Statistical analysis was carried out in GraphPad Prism version 9.1.0 for Windows GraphPad Software, San Diego, California USA, www.graphpad.com. Data are represented as mean ± standard error of the mean (s.e.m.). Non-parametric tests were performed in all cases. Number of samples (N) and the method used to test statistical significance are stated in each figure legend. Supplemental Table T3 contains further details of group comparisons and *P* values obtained.

## Supplemental Material

sj-docx-1-tej-10.1177_20417314221127908 – Supplemental material for Molecular and electrophysiological evaluation of human cardiomyocyte subtypes to facilitate generation of composite cardiac models Click here for additional data file. Supplemental material, sj-docx-1-tej-10.1177_20417314221127908 for Molecular and electrophysiological evaluation of human cardiomyocyte subtypes to facilitate generation of composite cardiac models by Jiuru Li, Alexandra Wiesinger, Lianne Fokkert, Bastiaan J. Boukens, Arie O. Verkerk, Vincent M. Christoffels, Gerard J.J. Boink and Harsha D. Devalla in Journal of Tissue Engineering

sj-xlsx-2-tej-10.1177_20417314221127908 – Supplemental material for Molecular and electrophysiological evaluation of human cardiomyocyte subtypes to facilitate generation of composite cardiac models Click here for additional data file. Supplemental material, sj-xlsx-2-tej-10.1177_20417314221127908 for Molecular and electrophysiological evaluation of human cardiomyocyte subtypes to facilitate generation of composite cardiac models by Jiuru Li, Alexandra Wiesinger, Lianne Fokkert, Bastiaan J. Boukens, Arie O. Verkerk, Vincent M. Christoffels, Gerard J.J. Boink and Harsha D. Devalla in Journal of Tissue Engineering

sj-xlsx-3-tej-10.1177_20417314221127908 – Supplemental material for Molecular and electrophysiological evaluation of human cardiomyocyte subtypes to facilitate generation of composite cardiac models Click here for additional data file. Supplemental material, sj-xlsx-3-tej-10.1177_20417314221127908 for Molecular and electrophysiological evaluation of human cardiomyocyte subtypes to facilitate generation of composite cardiac models by Jiuru Li, Alexandra Wiesinger, Lianne Fokkert, Bastiaan J. Boukens, Arie O. Verkerk, Vincent M. Christoffels, Gerard J.J. Boink and Harsha D. Devalla in Journal of Tissue Engineering

sj-xlsx-4-tej-10.1177_20417314221127908 – Supplemental material for Molecular and electrophysiological evaluation of human cardiomyocyte subtypes to facilitate generation of composite cardiac models Click here for additional data file. Supplemental material, sj-xlsx-4-tej-10.1177_20417314221127908 for Molecular and electrophysiological evaluation of human cardiomyocyte subtypes to facilitate generation of composite cardiac models by Jiuru Li, Alexandra Wiesinger, Lianne Fokkert, Bastiaan J. Boukens, Arie O. Verkerk, Vincent M. Christoffels, Gerard J.J. Boink and Harsha D. Devalla in Journal of Tissue Engineering
