# Supplementary material for: Molecular and electrophysiological evaluation of human cardiomyocyte subtypes to facilitate generation of composite cardiac models
Source: J Tissue Eng. 2022 Oct 18;13:20417314221127908. doi: 10.1177/20417314221127908 (PMC9583221; doi:10.1177/20417314221127908)
Supplement: sj-docx-1-tej-10.1177_20417314221127908 – Supplemental material for Molecular and electrophysiological evaluation of human cardiomyocyte subtypes to facilitate generation of composite cardiac models [file sj-docx-1-tej-10.1177_20417314221127908.docx]

**Molecular and Electrophysiological Evaluation of Human Cardiomyocyte Subtypes to Facilitate Generation of Composite Cardiac Models**

Jiuru Li^1^, Alexandra Wiesinger^1^, Lianne Fokkert^1^, Bastiaan J. Boukens^1^, Arie O. Verkerk^1,2^, Vincent M. Christoffels^1^, Gerard J.J. Boink^1,3,#^, Harsha D. Devalla^1,#^

^1^Department of Medical Biology, ^2^Department of Experimental Cardiology and ^3^Department of Cardiology

Amsterdam University Medical Centers, University of Amsterdam, Meibergdreef 9, 1105 AZ, Amsterdam, the Netherlands.

**Supplementary Table 1**

| **TARGET** | **SEQUENCE** |
| --- | --- |
| TNNT2 | F: TTCGACCTGCAGGAGAAGTT R: GCGGGTCTTGGAGACTTTCT |
| ACTN2 | F:AACCACTTTGACAGGAGGAAGAA R: CTTGCCCGTTGGGATCTACC |
| NXK2.5 | F: CCAAGGACCCTAGAGCCGAA R: ACACGTCTCACTCAGCATTTGT |
| SHOX2 | F: CCATAAAGGTGTTCTCATAGGGGC R: AACCTGAAAGGACAAGGGCG |
| TBX18 | F: TTGCTAAAGGCTTCCGAGAC R: AGGTGGAGGAACTTGCATTG |
| HCN4 | F: CCACTGCCCTCATCCAGTC R: GGTAGCGGTGCTCGTAGTAG |
| NR2F2 | F: CCGAGTACAGCTGCCTCAA  R: TTTTCCTGCAAGCTTTCCAC |
| NPPA | F: CGATCTGCCCTCCTAAAAAGC R: TTGTCCTCCCTGGCTGTTATC |
| KCNJ3 | F: TGTCGTCATCCTAGAAGGCA  R: AAAAACGATGACCCCAAAGA |
| MYH7 | F: AACACCAACCTGTCCAAGTTCC R: TGAGCAGATCAAGATGTGGCAA |
| MYL2 | F: TACGTTCGGGAAATGCTGAC R: TTCTCCGTGGGTGATGATG |
| HOPX | F: GGGCTGTTACAGAAGAAGACTGG R: GCTGCTTAAACCATTTCTGGGTC |
| SCN5A | F: TCACTCGCTCTTCAACATGC R: AGGTGTAAATGGCGGTGAAG |

**Supplementary Figure 1**


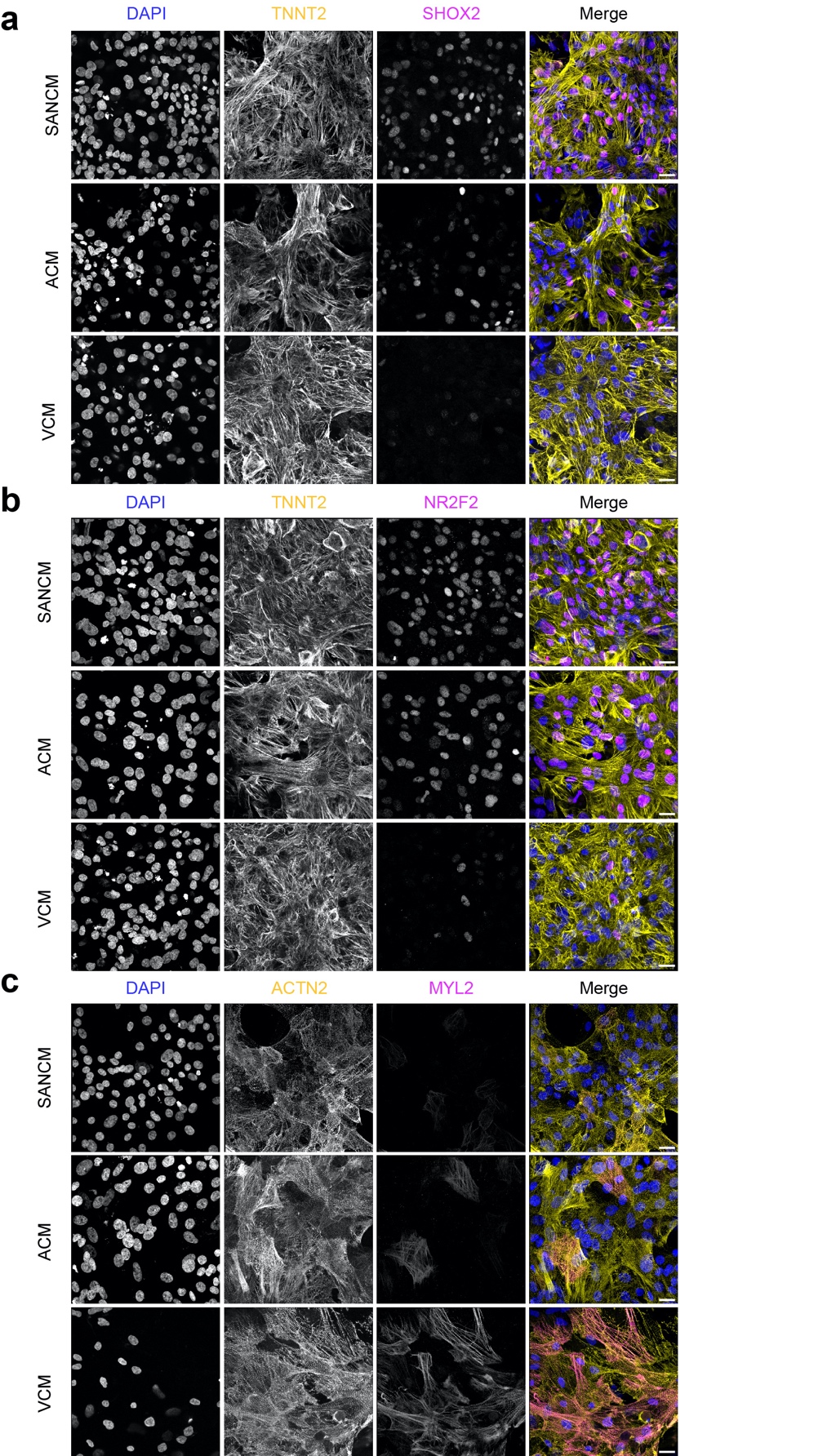


**­Supplementary Figure S1 (Related to Main Figure 1).** Immunofluorescence staining demonstrating the expression of SHOX2 and TNNT2 (a), NR2F2 and TNNT2 (b) and, MYL2 and ACTN2 (c), in SANCM, ACM and VCM. Scale bars, 20 μm.

**Supplementary Figure 2**
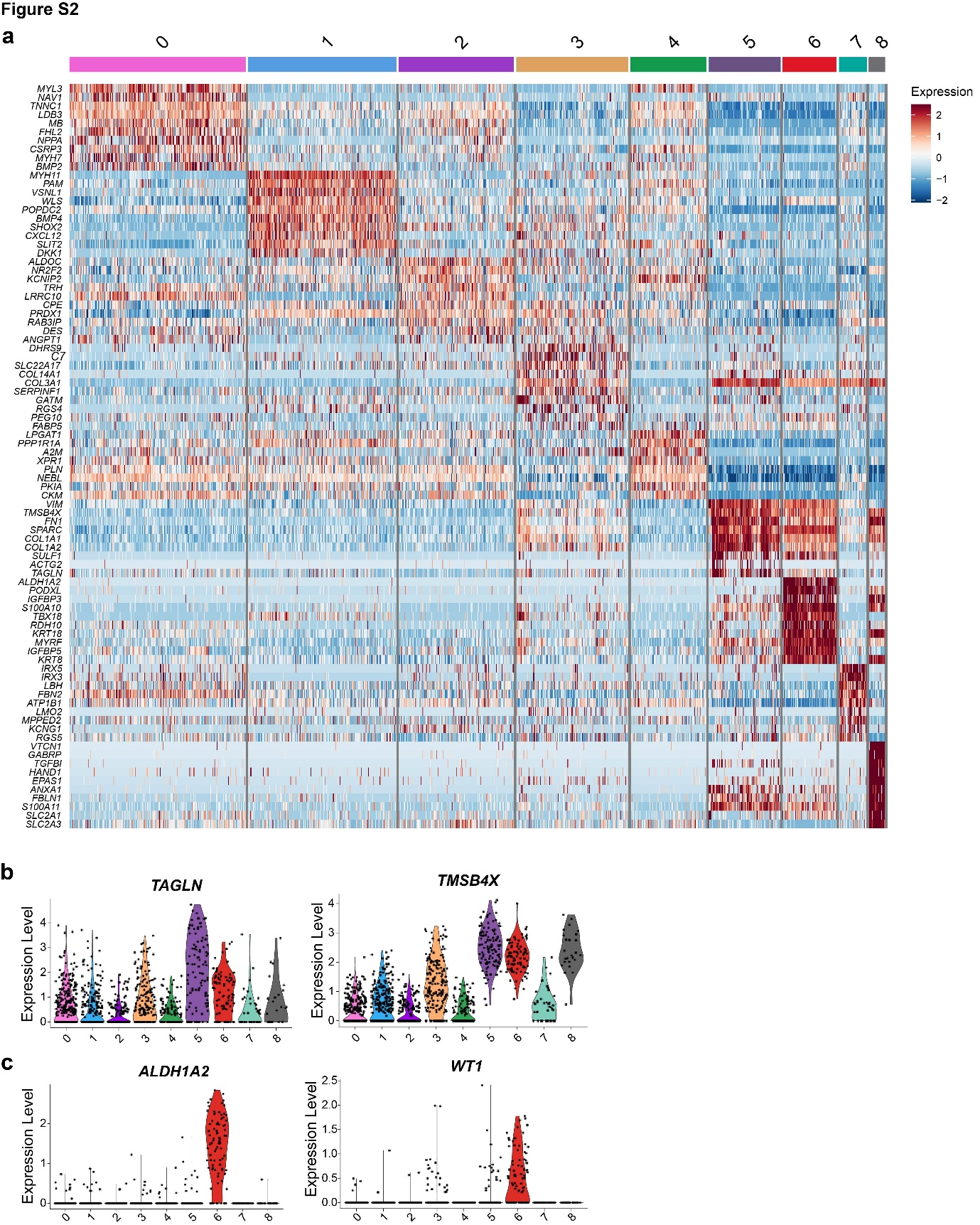


**Supplementary Figure S2 (Related to Main Figure 2).** Single cell RNA-sequencing analysis of day 19 SANCM and ACM cultures. (a) Heatmap showing top 10 differentially expressed genes of all clusters identified in SANCM and ACM cultures. (b-c) Violin plots depicting expression of smooth muscle cell-associated genes in cluster 5 (b) and proepicardial-associated genes in cluster 6 (c).

**Supplementary Figure 3**


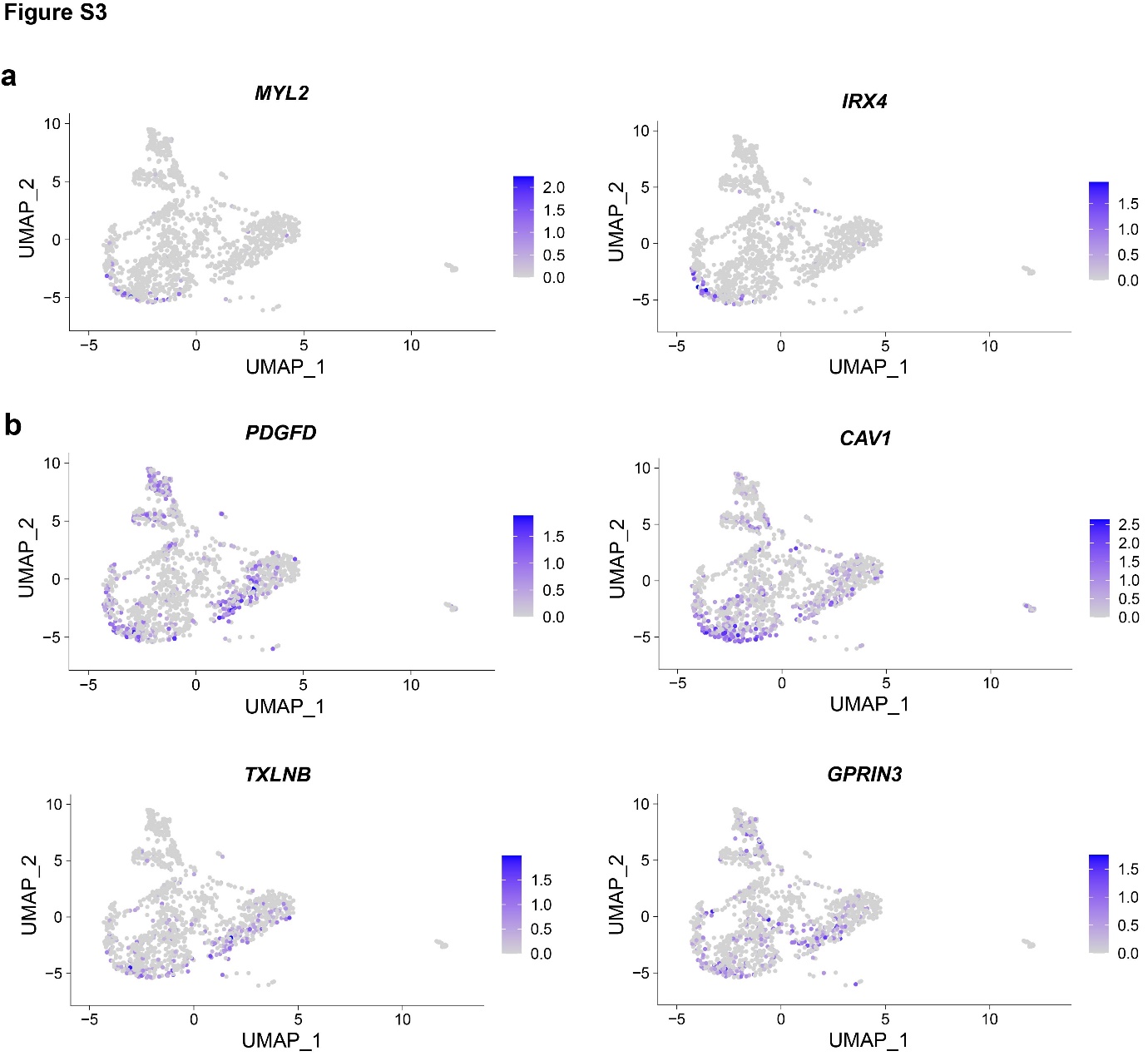


**Supplementary Figure S3 (Related to Main Figure 2).** Single cell RNA-sequencing analysis of day 19 SANCM and ACM cultures. (a) UMAP feature plots showing expression of *MYL2* and *IRX4* in a subpopulation of SANCM (SAN-transitional cells) that cluster with ACM in cluster 0. (b) expression of genes shared between SANCM present in clusters 1, 4 and a subpopulation of SANCM (SAN-transitional cells) that cluster with ACM in cluster 0.

**Supplementary Figure 4


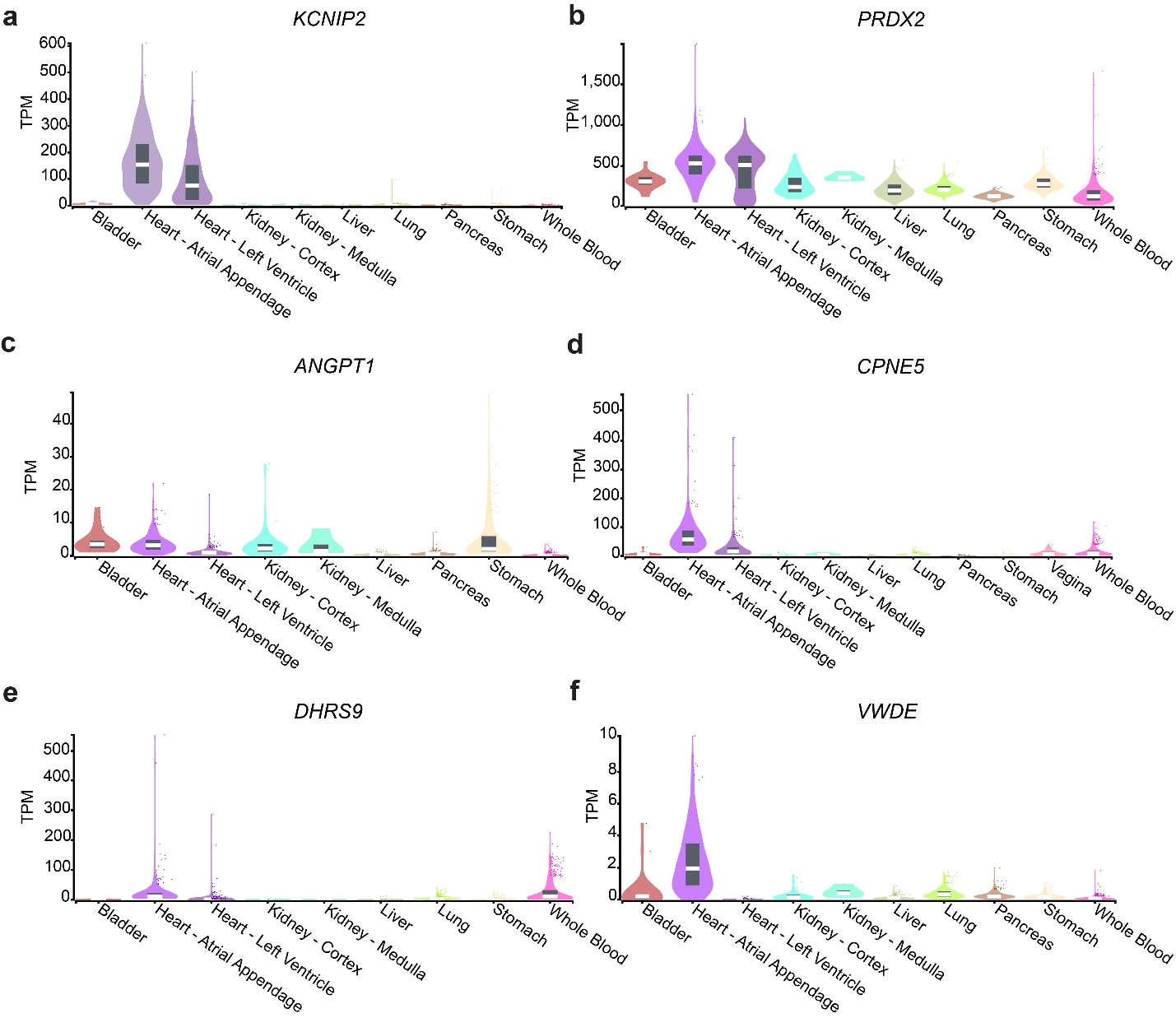
**

**Supplementary Figure S4 (Related to Main Figure 2).** Expression of genes (transcripts per kilobase million, TPM) identified in a subpopulation of ACM in human tissues. Preferential expression of (a) *KCNIP2* (b) *PRDX2* (c) *ANGPT1* (d) *CPNE5* (e) *DHRS9* and (f) *VWDE* in atrial versus ventricular tissue of human adult heart*.*

**Supplementary Figure 5**

**
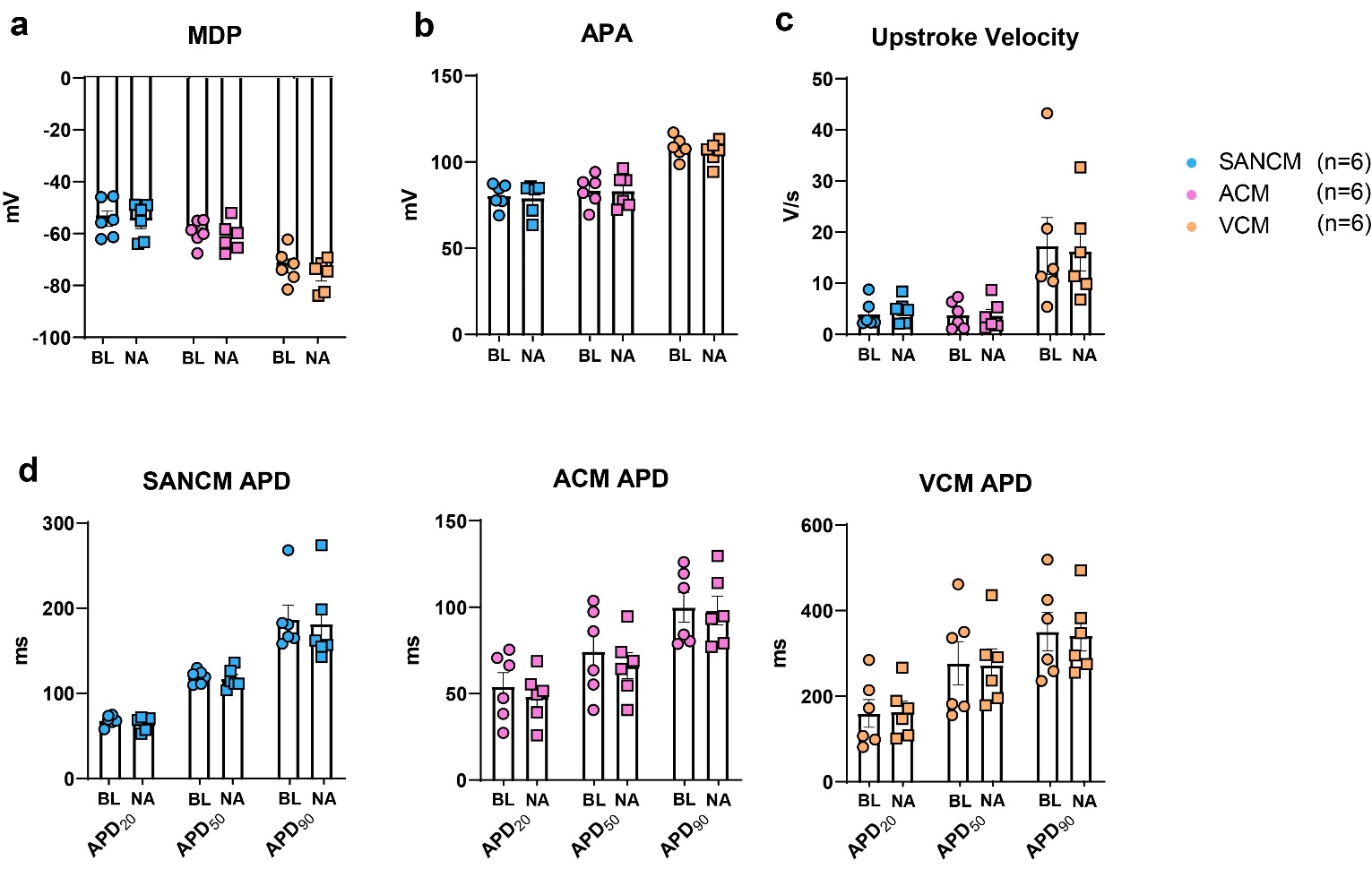
**

**Supplementary Figure S5 (Related to Main Figure 4).** Effects of 100 nM noradrenaline on action potential properties of cardiomyocyte subtypes. (a) Membrane diastolic potential, MDP, (b) maximum action potential amplitude, APA (c) Upstroke velocity, dV/dt_max_ (d) action potential duration at 20%, APD_20_, 50%, APD_50_, and 90% repolarization, APD_90_. N = 6 from four independent differentiations. Error bars, s.e.m.

**Supplementary Figure 6**

**
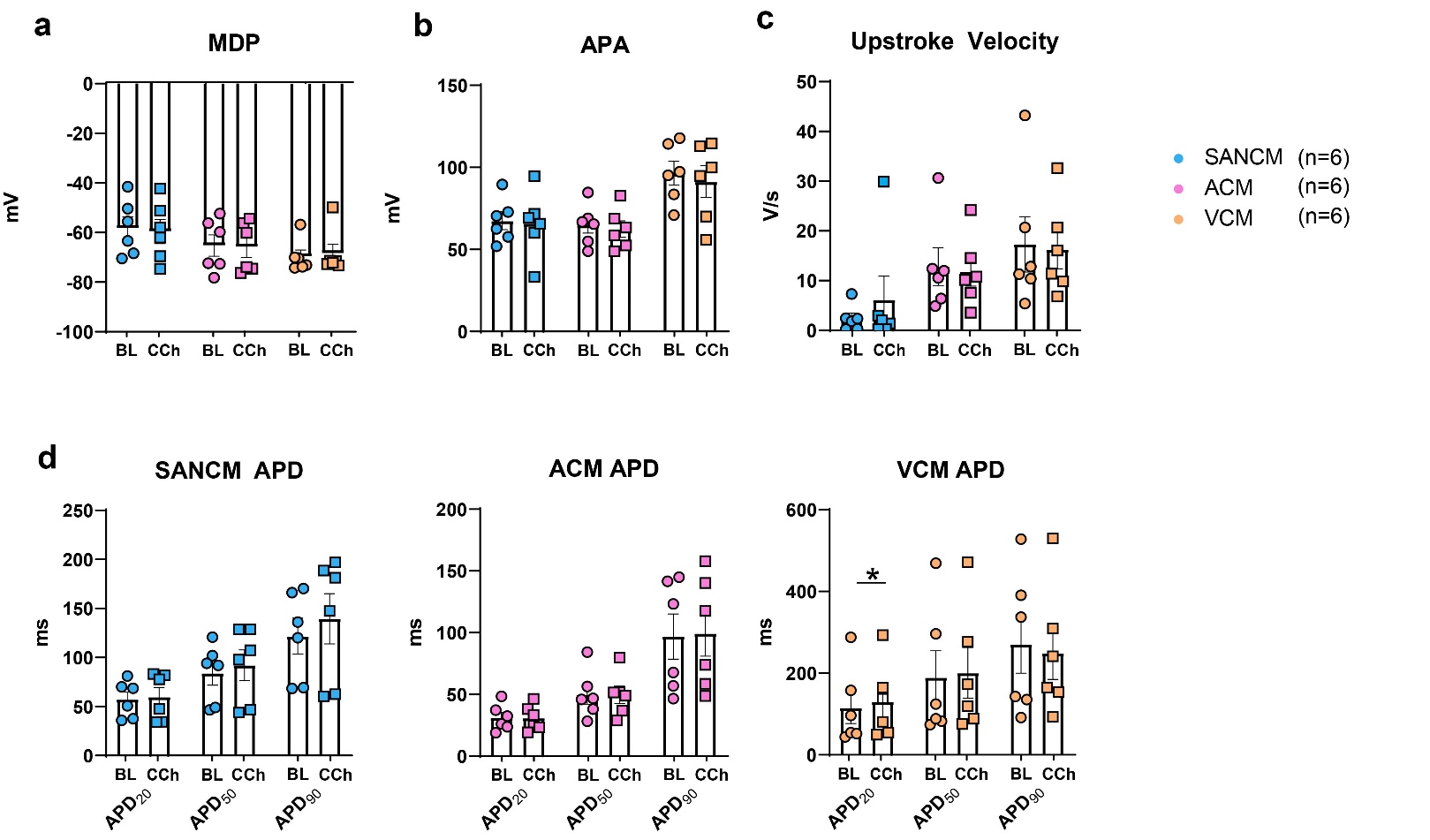
**

**Supplementary Figure S6 (Related to Main Figure 4).** Effects of 10 µM carbachol on action potential properties of cardiomyocyte subtypes. (a) Membrane diastolic potential, MDP, (b) maximum action potential amplitude, APA (c) Upstroke velocity, dV/dt_max_ (d) action potential duration at 20%, APD_20_, 50%, APD_50_ and 90% repolarization, APD_90_. N = 6 from four independent differentiations. Error bars, s.e.m. Wilcoxon’s Test. *P<0.05.

**Supplementary Figure 7


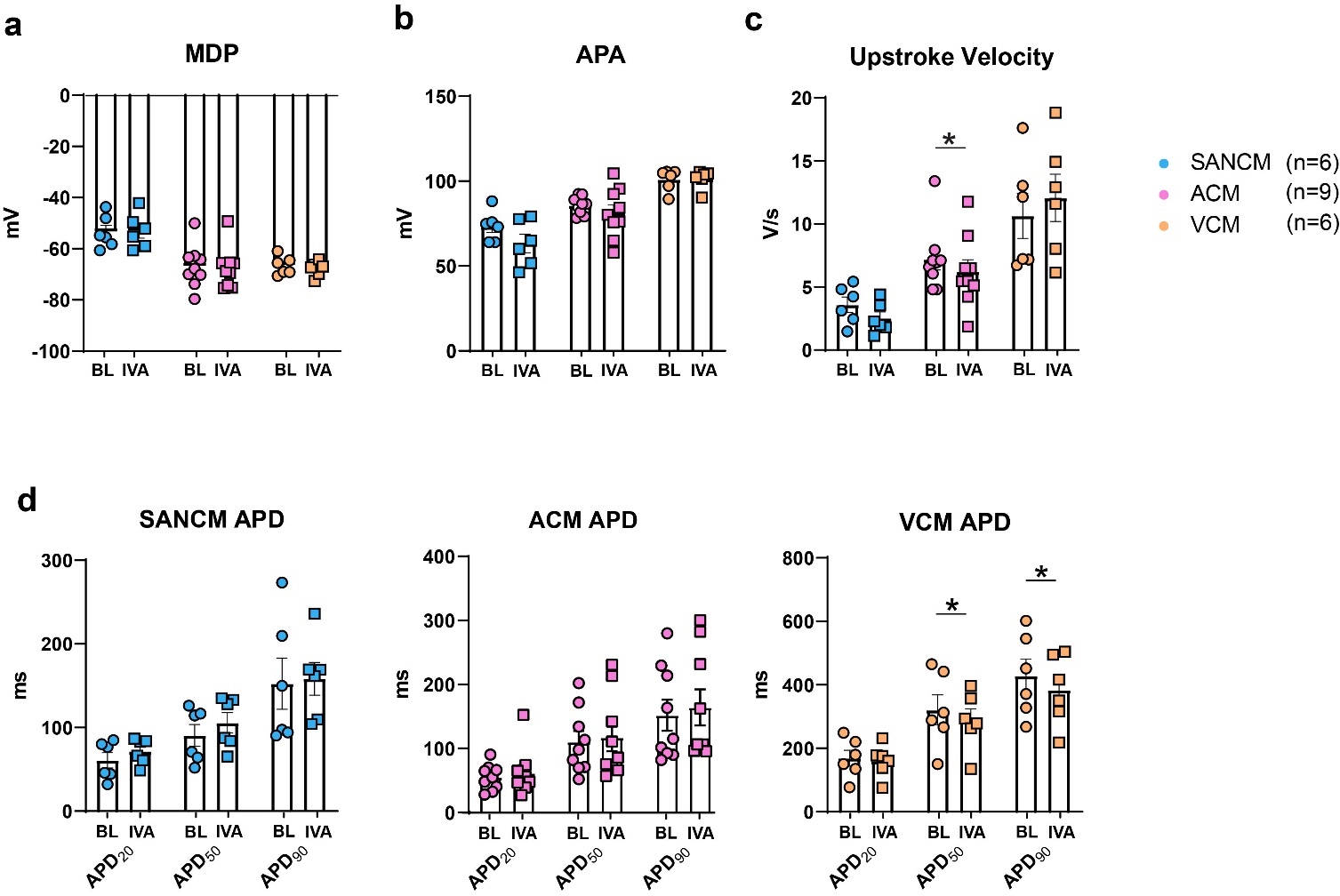
**

**Supplementary Figure S7 (Related to Main Figure 4).** Effects of 3 µM ivabradine on action potential properties of cardiomyocyte subtypes. (a) Membrane diastolic potential, MDP, (b) maximum action potential amplitude, APA (c) Upstroke velocity, dV/dt_max_: (d) action potential duration at 20%, APD_20_, APD_50_ and 90% repolarization, APD_90_. N = 6 from four independent differentiations, ACM; N = 9. Error bars, s.e.m. Wilcoxon’s Test. *P<0.05.

**Supplementary Figure 8**


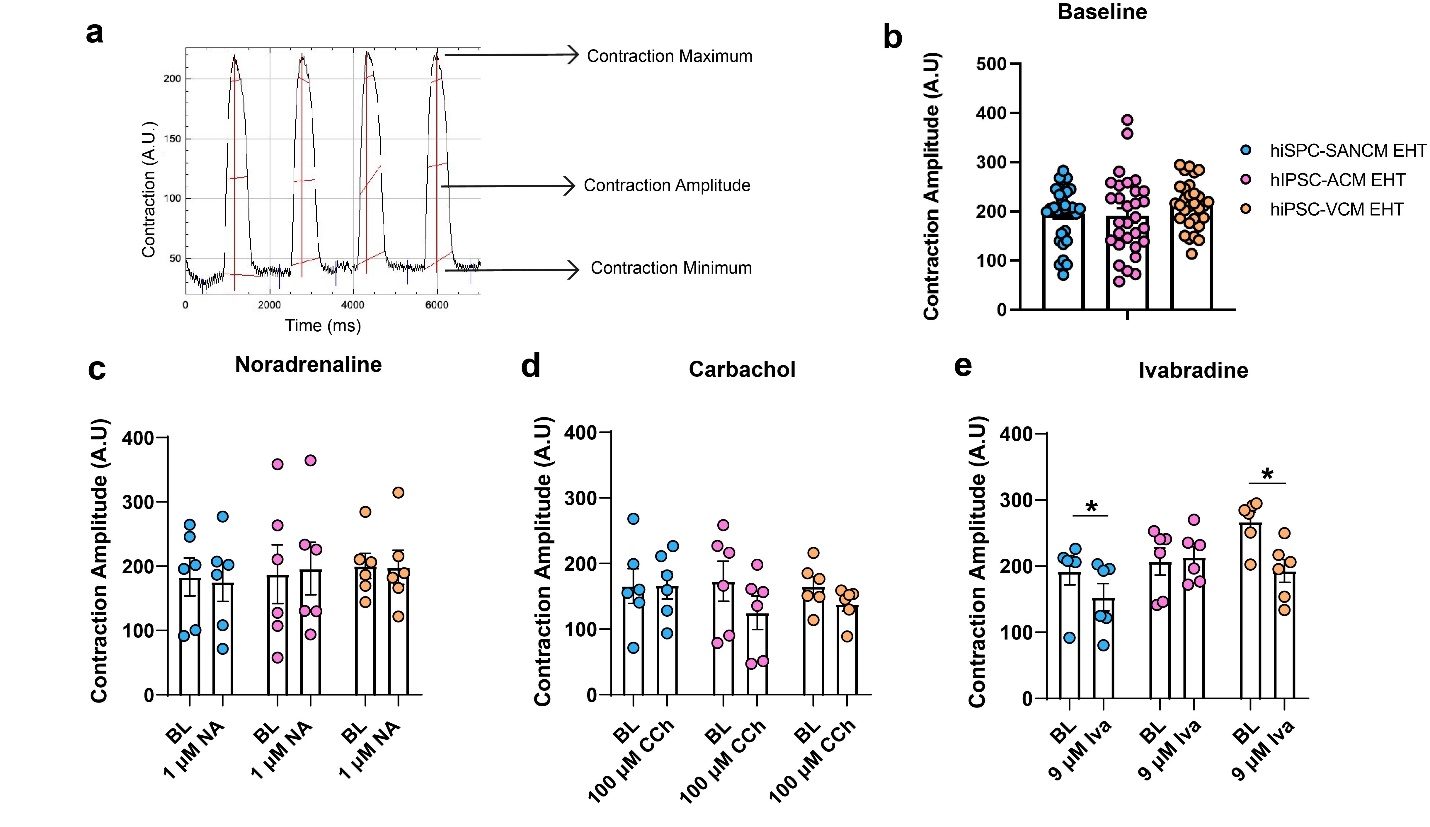
 **Supplementary Figure S8 (Related to Main Figure 6).** Contraction properties of EHT subtypes. Contraction analysis performed using Musclemotion^TM^ (a) illustration of an example trace showing contraction amplitude computed from contraction maximum and contraction minimum values (b) Contraction amplitudes in EHT subtypes, n = 30 EHTs/group (c - e), contraction amplitude at baseline and in response to 1 µM noradrenaline (c) 100 µM carbachol (d) 9 µM ivabradine (e) N = 6 EHTs/subtype from four independent differentiations. Error bars, s.e.m. Pairwise error calculated with Wilcoxon’s Test. Multiple group comparison with Kruskal Wallis Test followed by Mann Whitney U-test for post hoc comparison, *P<0.05.
